# The RSC (Remodels the Structure of Chromatin) complex of *Candida albicans* shows compositional divergence with distinct roles in regulating pathogenic traits

**DOI:** 10.1371/journal.pgen.1009071

**Published:** 2020-11-05

**Authors:** Vinutha K. Balachandra, Jiyoti Verma, Madhu Shankar, Timothy M. Tucey, Ana Traven, Ralf B. Schittenhelm, Santanu K. Ghosh

**Affiliations:** 1 IITB-Monash Research Academy, Indian Institute of Technology Bombay, Mumbai, India; 2 Infection and Immunity Program and the Department of Biochemistry and Molecular Biology, Biomedicine Discovery Institute, Monash University, Clayton, Victoria, Australia; 3 Monash Proteomics & Metabolomics Facility, Biomedicine Discovery Institute, Monash University, Clayton, Victoria, Australia; 4 Department of Biosciences and Bioengineering, Indian Institute of Technology, Bombay, Mumbai, India; University of Kent, UNITED KINGDOM

## Abstract

Regulation of gene expression programs is crucial for the survival of microbial pathogens in host environments and for their ability to cause disease. Here we investigated the epigenetic regulator RSC (Remodels the Structure of Chromatin) in the most prevalent human fungal pathogen *Candida albicans*. Biochemical analysis showed that CaRSC comprises 13 subunits and contains two novel non-essential members, which we named Nri1 and Nri2 (Novel RSC Interactors) that are exclusive to the CTG clade of *Saccharomycotina*. Genetic analysis showed distinct essentiality of *C*. *albicans* RSC subunits compared to model fungal species suggesting functional and structural divergence of RSC functions in this fungal pathogen. Transcriptomic and proteomic profiling of a conditional mutant of the essential catalytic subunit gene *STH1* demonstrated global roles of RSC in *C*. *albicans* biology, with the majority of growth-related processes affected, as well as mis-regulation of genes involved in morphotype switching, host-pathogen interaction and adaptive fitness. We further assessed the functions of non-essential CaRSC subunits, showing that the novel subunit Nri1 and the bromodomain subunit Rsc4 play roles in filamentation and stress responses; and also interacted at the genetic level to regulate cell viability. Consistent with these roles, Rsc4 is required for full virulence of *C*. *albicans* in the murine model of systemic infection. Taken together, our data builds the first comprehensive study of the composition and roles of RSC in *C*. *albicans*, showing both conserved and distinct features compared to model fungal systems. The study illuminates how *C*. *albicans* uses RSC-dependent transcriptional regulation to respond to environmental signals and drive survival fitness and virulence in mammals.

## Author summary

The nucleosomal structure of chromatin poses a natural barrier to transcription. As such, a host of factors regulate transcription by modulating the dynamic accessibility of DNA to the transcriptional machinery. One such factor, the Remodels the Structure of Chromatin (RSC) complex, disrupts histone-DNA contacts and causes DNA translocation using energy derived from ATP hydrolysis. The importance of gene expression in regulating pathogenic traits has been widely recognized in the pathogenic yeast *Candida albicans*, however the contributions of chromatin regulators in general, and the RSC complex in particular remain understudied. Here, we show that while the *C*. *albicans* RSC has broadly conserved counterparts in the model yeasts, it has distinct composition and functions. We identified two novel RSC subunits specific to the CTG clade of fungi, one of which is required for proper filamentation and adaptive fitness of the pathogen. Perturbation of RSC function led to the misregulation of genes controlling pathogenic attributes, and attenuated the virulence of *C*. *albicans* in a mammalian infection model. Our findings highlight the species-specific distinctions of RSC in the pathogenic landscape of *C*. *albicans*, with implications for targeting this complex for designing antifungal interventions in the future.

## Introduction

*Candida* spp. account for over 400,000 cases of invasive infections per year globally [1]. Of these species, *Candida albicans* is the most prevalent cause of both invasive and superficial human fungal infections [1, 2]. This organism, which otherwise exists as a commensal in healthy human hosts, can cause high rates of fatal infections under immunocompromised conditions. Inside the host, *C*. *albicans* is challenged by hostile conditions such as immune responses, metabolic and oxidative stresses and exposure to anti-fungal drugs during therapy. To counter these stresses, *C*. *albicans* has evolved robust adaptive response mechanisms such as expression of proteins that facilitate tissue adhesion and invasion, host cell damage, acquisition and utilization of diverse carbon sources, and adaptation to various environmental assaults (reviewed in [3]). Additionally, *C*. *albicans* undergoes a morphology switch from yeast to hyphal form in response to a range of external signals [4], and this transition is regarded as an important attribute of virulence since it enables tissue adhesion and invasion [5], escape from immune cells [6] and biofilm formation [7].

Mechanistically, the modulation of transcriptional programs lies at the core of all these adaptive responses. However, the nucleosomal structure of the chromatin poses a natural barrier to transcription and, two general classes of epigenetic regulators are important for modulating the dynamic accessibility of DNA to the transcriptional machinery–chromatin modifiers and chromatin remodeling complexes (CRCs). Unlike chromatin modifiers that covalently alter the nucleosome structure, the CRCs function by destabilizing histone-DNA contacts and effect DNA translocation using energy derived from ATP hydrolysis [8]. The functions of the CRCs in relation to the pathogenic attributes of *C*. *albicans* are primarily known from the studies on the SWI/SNF complex [9–12]. However, how other CRCs feed into these pathways to regulate *C*. *albicans* fitness and virulence in the host, is poorly understood. In this context, the only CRC reported to be essential for growth in the model yeasts- the Remodels the Structure of Chromatin (RSC) complex draws considerable attention.

RSC is a member of the SWI/SNF family of chromatin remodelers which is at least ten times more abundant than the SWI/SNF complex [13]. Structurally, this complex is composed of a main motor/catalytic subunit harboring ATPase activity, a pair of actin-related proteins (ARPs), a set of core subunits, and a set of accessory subunits that are organism-specific [14]. The RSC complex in the model fungus *Saccharomyces cerevisiae* (ScRSC) consists of 17 subunits with Sth1 as the catalytic subunit [13]. Two isoforms of the ScRSC exist which are distinguished by the presence of either Rsc1 or Rsc2 [13, 15]. The *Schizosaccharomyces pombe* RSC (SpRSC) is considered to be more similar in composition to the metazoan RSC than to the ScRSC, as it lacks several of the ScRSC components but harbors an Arp4-like subunit (Arp42) observed in metazoan [16]. While, the catalytic subunit of RSC is essential for growth in yeast [13, 17], fly [18, 19], mouse [20] and human [21], characterization of RSC orthologs in *Arabidopsis thaliana* [22] and the basidiomycete *Cryptococcus neoformans* [23] indicates otherwise. Nevertheless, RSC is believed to be crucial for gene expression in various eukaryotes as it impacts transcription mediated by all three RNA polymerases [24] and regulates nucleosome density at both promoter [25, 26] and coding sequences [27]. However, the effect of RSC mutations on gene expression is different for different subunits and rarely conserved across organisms. For example, genes encoding ribosomal proteins are induced in *rsc30ΔΔ* but repressed in the temperature sensitive mutants of *S*. *cerevisiae RSC3* and *RSC4* [28, 29]. Furthermore, mutation of the *ScRSC4* subunit significantly changes the transcriptional profile of RNA polymerase II, which is in stark contrast to the modest transcriptional changes observed after *SpRSC4* deletion [16, 29]. Besides transcriptional regulation, the yeast RSC complexes play vital roles in cell cycle, with inactivation of RSC in these organisms leading to a G2-M arrest [13, 30]. Remarkably, cell cycle progression in *Drosophila melanogaster* does not depend on the DmRSC (PBAP), but on the DmSWI/SNF complex (BAP), which controls entry into mitosis [31]. Thus, despite being conserved from yeast to higher eukaryotes, the distinct composition of RSC in diverse eukaryotic species might vouch for its various regulatory roles in species-specific biology.

The functions of RSC in *C*. *albicans* (CaRSC) have remained understudied, and at present its subunit composition and requirements for virulence are unknown. The only known roles come from a previous study done by this group showing roles of CaRSC in chromosome segregation using a conditional mutant of *STH1* [32]. Here, we combined biochemical, genetic and “omics” approaches to comprehensively investigate the RSC complex in *C*. *albicans*. We demonstrate that this complex is distinct from RSC in other model fungi and contains two additional subunits with no apparent homologs present in distantly related species. Furthermore, loss of CaRSC function led to mis-regulation of various pathogen-specific attributes in addition to growth-related processes, and phenotypic analysis of RSC mutants showed conditional defects in morphogenesis and stress responses of *C*. *albicans*. Finally, we show that the virulence of a RSC mutant is compromised in a murine systemic infection model. Collectively, our findings position the RSC complex as a determinant in the midst of the pathogenic landscape of *C*. *albicans* and make the complex an attractive target for anti-fungal drug discovery in the future.

## Results

### The CaRSC is compositionally and genetically distinct from ScRSC and contains two novel subunits

**Fig 1:**
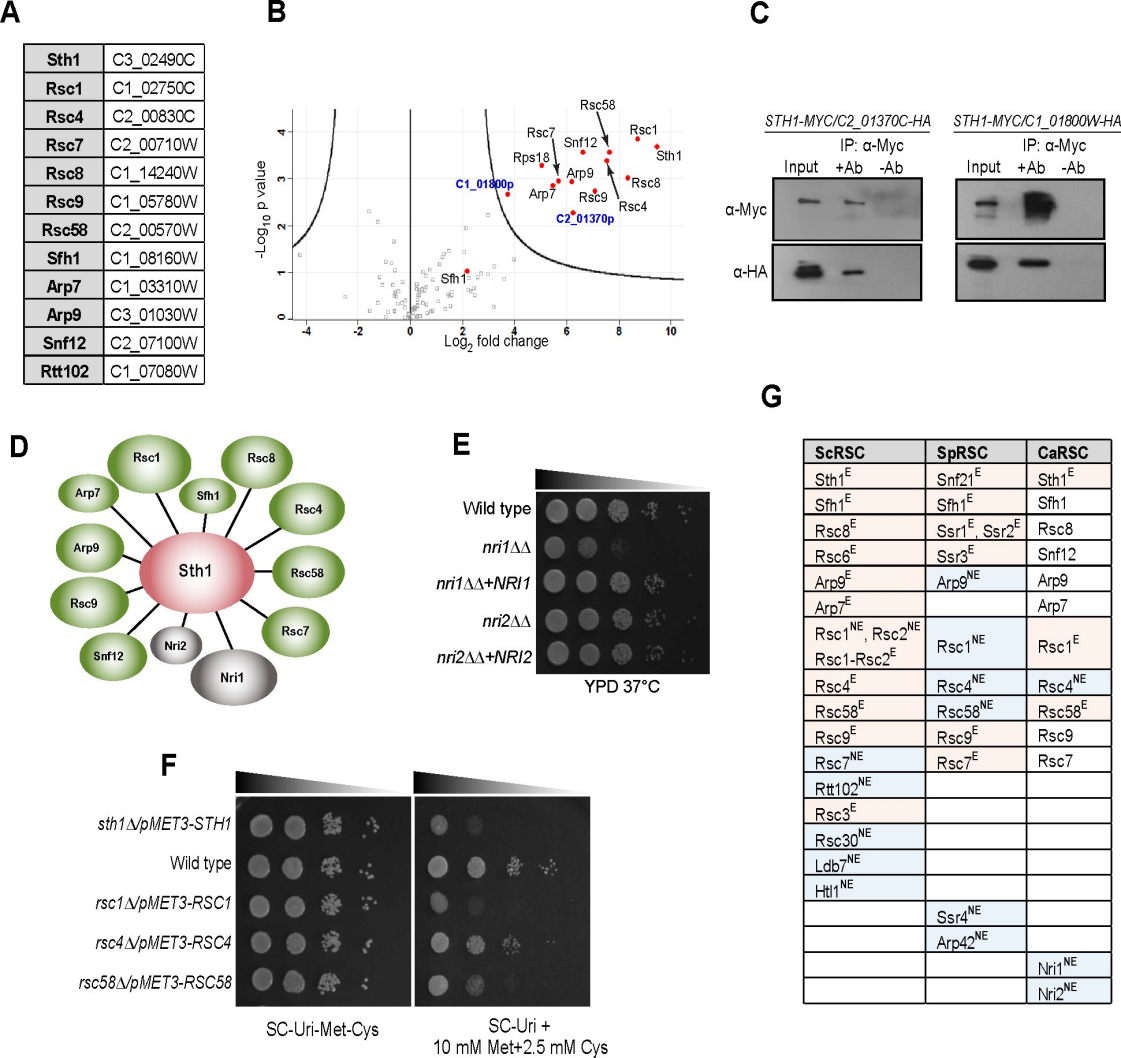
*The C. albicans* RSC complex is compositionally and genetically distinct from RSC of model yeasts. **A.** List of putative RSC subunits and their respective ORFs in *C*. *albicans* predicted from the Candida Genome Database. **B.** Volcano plot representation of significantly enriched proteins that copurified with Sth1-Myc (SGC2003) compared to a no-tag control (SGC2006). Significant interactors identified are shown on the right side of the parabola as red dots, except for one potential interactor with low confidence score (Sfh1), highlighted on the left side. The grey boxes represent the potential background interactors. The two novel interactors are labelled in blue. The x-axis denotes the enrichment of the interaction in Sth1-Myc samples with respect to no-tag control, represented as a natural logarithm of normalized intensity values. The negative logarithm to the base 10 of the p-value, based on Student’s *t-*test, is given on the y-axis. **C.** Cell lysates from cells expressing Sth1-Myc and C2_01370p-HA (SGC2043) or Sth1-Myc and C1_01800p-HA (SGC2044) were immunoprecipitated with the polyclonal anti-myc (ab9106) antibody. Sth1-Myc and C2_01370p-HA or C1_01800p-HA were detected by immunoblotting using monoclonal anti-Myc (9E10) and anti-HA (3F10) antibodies respectively. Two percent of input and 50% of eluate were loaded. Uncropped images of blots shown here are presented in S8 Fig. **D.** Schematic model of the *C*. *albicans* RSC complex based on AP-MS of Sth1-Myc. Sth1, the catalytic RSC component is shown in red, while the conserved subunits and the newly identified interactors are shown in green and grey, respectively. The size of the ellipses approximately represents the size of the respective proteins. The length of the lines connecting Sth1 to other RSC subunits are drawn arbitrarily. **E.** Growth of wild type (SGC2024), *nri1ΔΔ* (SGC2047) and *nri2ΔΔ* (SGC2032) mutants, and their corresponding re-integrant (*nri1*ΔΔ+*NRI1*:SGC2049; *nri2*ΔΔ+*NRI2*:SGC2036) strains on YPD plates, incubated at 37°C and photographed after 24 hours. **F.** Essentiality of *pMET3*-based conditional RSC mutants of *C*. *albicans* (*MET3-STH1*:SGC65; *MET3-RSC1*:SGC2070; *MET3-RSC4*:SGC2053; *MET3-RSC58*:SGC2075). Serial dilutions of the indicated strains were spotted on growth permissive (SC-Uri-Met-Cys) and repressive (SC-Uri+10 mM Met+2.5 mM Cys) plates and incubated at 30°C for 2 days. Since these strains were constructed in SN148 background using *URA3* marker, SN148-*URA3* (SGC2006) was used as the wild type control. **G.** Comparison of RSC composition and subunit essentiality (E-essential and NE-non-essential colored in red and blue respectively) in *S*. *cerevisiae*, *S*. *pombe* and *C*. *albicans*.

Homology based searches for CaRSC subunits in the CGD (Candida Genome Database) based on *S*. *cerevisiae* data returned ORFs annotated as putative components of the CaRSC (Fig 1A and [32]). Identifiable homologs of the ScRSC subunits Rsc3, Rsc30, Htl1 and Ldb7 could not be retrieved for *C*. *albicans* from the CGD. In addition, only one of the paralogous subunits, Rsc1 and Rsc2, was identified in *C*. *albicans* based on homology. To precisely determine the composition of the CaRSC complex, we immunopurified Myc-tagged Sth1, the core catalytic subunit of the CaRSC complex encoded by C3_02490C [32], and identified its physical interactors using high-resolution tandem mass spectrometry (LC-MS/MS). Wild-type cells expressing untagged Sth1 served as negative control, and all immunoprecipitation experiments were analyzed in biological triplicates. We identified 95 proteins across all samples falling below a false discovery rate (FDR) threshold of 1% (S1 Table). With the exception of Sfh1 and Rtt102, all bonafide RSC complex subunits were identified and quantified with significantly elevated levels in the Sth1-Myc immunoprecipitation samples (Fig 1B, S1 Table). However, a closer inspection revealed that Sfh1 was present among the background proteins (Fig 1B) and was not observed to be significantly enriched due to its identification in only two of the three analyzed replicates. In addition, Sfh1 was not identified in any control sample unlike other background proteins (see AP-MS Raw data-LFQ intensities in S1 Table). Thus, our biochemical data closely aligned with the homology-based results except for Rtt102, which was predicted (Fig 1A) but not identified in our experiment (Fig 1B). Strikingly, we identified two novel hitherto uncharacterized proteins that co-purified with Sth1—C2_01370p and C1_01800p, whose homologs are annotated only in the CTG clade of fungi, which contains several important *Candida* species in addition to *C*. *albicans*. We confirmed these results by co-immunoprecipitating HA-tagged versions of these novel interactors with Sth1-Myc (Fig 1C). Since examination of their primary sequences suggested that neither of these proteins are conserved in *S*. *cerevisiae*, *S*. *pombe*, *C*. *glabrata* or in metazoans, we named them Nri1 (C2_01370p) and Nri2 (C1_01800p) for Novel RSC Interactors. The interactions were further validated by reciprocal co-immunoprecipitations of Myc-tagged Nri1 and Nri2 followed by LC-MS/MS, which found most of the RSC subunits identified in this study co-purifying with both Nri1 and Nri2 (S2 Table). Taken together, these data indicate that the CaRSC contains two novel CTG-clade-specific proteins, Nri1 and Nri2, in addition to 11 conserved subunits (Fig 1D).

*NRI1* and *NRI2* are non-essential for viability in *C*. *albicans*, as evident from analyses of their homozygous mutants (Fig 1E). The *nri1*ΔΔ mutant has a fitness defect under standard growth conditions (S1A and S1B Fig). To test the requirement of conserved CaRSC subunits for viability, we focused on *RSC1*, *RSC4* and *RSC58* that encode bromodomain-containing subunits—all of which are essential in *S*. *cerevisiae* (*rsc1*^*-*^*rsc2*^*-*^ is inviable) but not in *S*. *pombe* [16]. Using repressible conditional mutants under the *MET3* promoter (repressible by methionine and cysteine), we show that while *RSC1* is essential in *C*. *albicans* to the same extent as the catalytic subunit *STH1*, *RSC4* is not (Fig 1F). Immunopurification of Sth1-Myc in *rsc4*ΔΔ background showed that the composition of CaRSC is unaffected in the absence of Rsc4 (S2 Fig, S1 Table). Among the bonafide RSC interactors, we also detected ribosomal proteins (Fig 1B and S2 Fig). However, these proteins along with histones, elongation factors and heat shock proteins are commonly identified as non-specific interactors in AP-MS experiments [33]. Hence, these are unlikely to be true RSC interactors. The growth of CaRsc58-depleted cells was also severely retarded (Fig 1F) suggesting essential or near-essential functions for *C*. *albicans RSC58*. Thus, although the CaRSC complex appears largely conserved as observed in other model fungi, our findings suggest divergence at the compositional and genetic level compared to *S*. *cerevisiae* and *S*. *pombe* (Fig 1G).

### Transcriptomic and proteomic analysis of loss of RSC function in *C*. *albicans*

Since our biochemical and genetic analyses revealed species-specific distinctions in the CaRSC complex (Fig 1G), we next addressed the global functions of RSC in *C*. *albicans* by analyzing the changes in the transcriptome and proteome of cells lacking the catalytic subunit Sth1, which can be considered as a “loss of RSC function” mutant. We generated a conditional mutant harboring a doxycycline (dox)-repressible allele of *STH1* in a hemizygous *STH1* mutant (*sth1*Δ*/pTetO-STH1-MYC;* S3A Fig). Sth1-Myc levels during dox-based depletion across various time points were monitored by immunoblotting (S3B Fig). Based on these results, a dox treatment period of 7 hours was chosen for both the transcriptomic and proteomic analysis as (i) cells remained in the yeast form, which allowed us to study Sth1 depletion in the absence of morphogenetic changes that occur upon prolonged depletion of Sth1 (S3C Fig) and (ii) cells were able to resume growth upon removal of dox (S3D Fig) suggesting that they did not lose viability. The effect of dox alone on gene expression, assessed using a near-isogenic *sth1*Δ*/STH1-MYC* strain, was found to be negligible at both RNA and protein levels (S3 Table, S4 Table).

**Fig 2:**
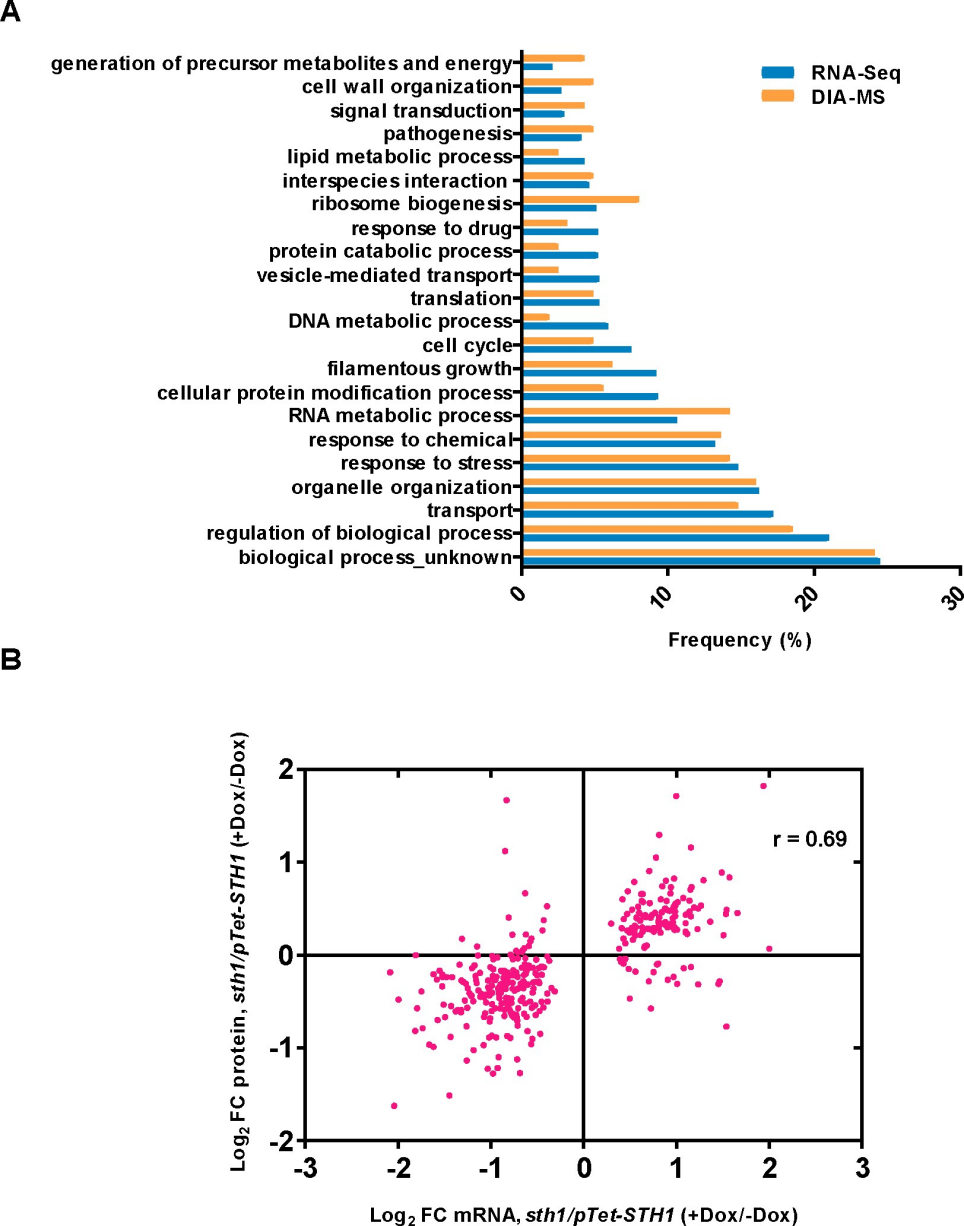
*sth1* mutant gene expression profiles show global roles of RSC in *C*. *albicans*. **A**. Sth1-dependent biological processes inferred by Gene Ontology (GO) analysis of differentially expressed genes (RNA-Seq, blue bars) and proteins (DIA-MS, orange bars). Y-axis represents the various GO terms of ‘biological process’, mapping to the expression dataset of *sth1/pTet-STH1* (SGC2001), while the x-axis denotes the frequency with which a specific GO term mapped to the candidate list provided. Raw data underlying graph given in S7 Table. **B.** Scatter plot depicting the relationship between the RNA-Seq (transcriptomic) and DIA-MS (proteomic) datasets of *sth1/pTet-STH1* (SGC2001) mutant. Pink dots in the plot represent proteins identified with their corresponding genes in the RNA-Seq dataset. The log2 FC (fold change) values of mRNAs (5% FDR) along with their corresponding protein log2 fold change values (5% FDR) are represented on x-axis and y-axis, respectively. The Pearson’s correlation coefficient (*r*) is indicated on the top right corner of the plot. Raw data underlying graph given in S7 Table.

Sth1 depletion led to substantial changes in both the transcriptome (567 up- and 692 down-regulated transcripts based on RNA-Seq; S3 Table) and the proteome (55 up- and 107 down-regulated proteins based on DIA-MS ((Data Independent Acquisition-Mass Spectrometry); S4 Table), considering a fold-change cutoff of 1.5 and a FDR threshold of 0.05. Gene ontology (GO) analysis (Fig 2A) revealed that the differentially expressed genes and proteins are involved in a variety of biological processes including “transport”, “organelle organization”, “stress response”, “RNA metabolic process”, “filamentous growth”, “translation” and “pathogenesis”. A good correlation was observed between the changes of the mRNA levels and their associated proteins (Pearson r = 0.69, N = 356, p-value<0.0001, Fig 2B), which is also evident from the similar GO terms mapping to the mis-regulated genes and proteins (Fig 2A).

**Fig 3:**
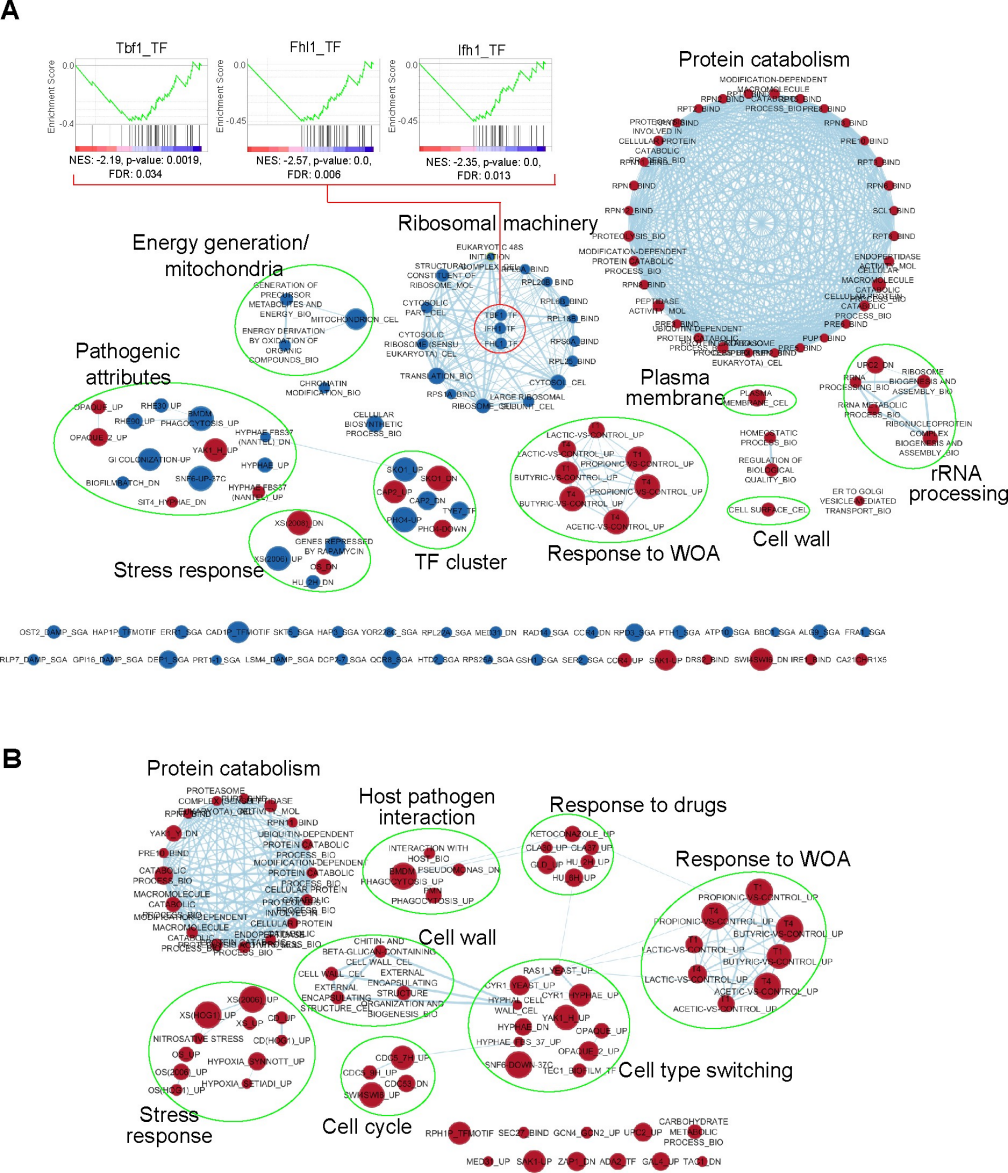
Enrichment analysis of the transcriptome and proteome of cells compromised in CaSth1 function. GSEA results of *sth1/pTet-STH1* transcriptome (**A**) and proteome (**B**) profiles were visualized using Enrichment Map function of Cytoscape. In **A**, GSEA plots representing enrichment of targets of Tbf1, Fhl1 and Ifh1 transcription factors (red ellipse), are shown above the enrichment map. The targets were enriched among the downregulated candidates of *sth1/pTet-STH1*, as indicated by a negative enrichment score on y-axis. The black lines on the x-axis represent individual genes which are ranked by expression values along a color scale, where blue represents downregulated and red denotes upregulated candidates (NES: Normalized Enrichment Score; FDR: False Discovery Rate). In the enrichment maps of **A** and **B**, the size of each solid circle (gene set) is proportional to the number of differentially expressed genes in that category and various such circles are connected by blue lines based on the number of overlapping genes. Gene sets corresponding to the down-regulated candidates are shown in blue circles while the red circles map to the up-regulated group. Clusters of gene sets that map to a common broader category are labelled and circled in green (WOA - Weak Organic Acids, TF - Transcription Factor).

Detailed inspection of Sth1-dependent genes and proteins was further carried out using GSEA (Gene Set Enrichment Analysis; S5 Table) and represented as a network of significantly enriched gene and protein clusters (Fig 3A and 3B). In agreement with the essential functions of RSC, we observed mis-regulation of various growth-associated processes in the transcriptomic and proteomic analyses of *sth1*. Genes encoding the ribosomal machinery were repressed in the *sth1* transcriptome (Fig 3A) and consistent with this observation, we further noticed the downregulation of several target genes of the transcription factors Tbf1, Ifh1 and Fhl1 (Fig 3A, shown at center of “ribosomal machinery” cluster), which largely control the expression of ribosomal protein genes [34]. Likewise, gene sets associated with “energy generation/mitochondria” and “chromatin modification” were also found to be repressed in the *sth1* transcriptome. Consistent with the repression of mitochondrial genes in *sth1*, we observed pronounced defects in the mitochondrial morphology of Sth1-depleted cells (S4 Fig). In addition, several gene sets corresponding to various stress responses and pathogen-specific traits (such as hyphae formation, white-opaque cell switch, biofilm formation, host-*Candida* interactions) were observed to be enriched in the *sth1* mutant. The *sth1* mutant transcriptome also shared similarity with the gene expression profiles of cells lacking specific transcription factors (TF) (Fig 3A; TF cluster) suggesting that CaRSC may act through modulating the DNA-binding activity or expression of these TFs.

The up-regulated transcripts mainly mapped to genes involved in protein catabolism, rRNA processing, response to weak organic acids as well as in the architecture of the cell wall and plasma membrane (Fig 3A), and a somewhat similar pattern was revealed by GSEA of the *sth1* mutant proteome (Fig 3B). Further proteome level analysis of *sth1* cells also revealed the upregulation of proteins involved in responses to (i) host-pathogen interaction (e.g. phagocytosis by bone-marrow derived macrophages and polymorpho nuclear leukocytes), (ii) drugs (e.g. Hsp90 inhibitor geldanamycin, anti-fungal drug ketoconazole and DNA synthesis inhibitor hydroxyurea), (iii) cell type switching (e.g. hyphae formation, white-opaque switch, biofilm formation) and (iv) various other stresses including hypoxia (Fig 3B).

Since most of these processes are modulated by specific transcriptional regulators, we analyzed the *sth1* transcriptome using PathoYeastract to predict TFs that may be regulated by Sth1 and in extension by the entire RSC complex in *C*. *albicans* (S5 Fig). In support of our GSEA results, we identified several TFs involved in the regulation of filamentation (Nrg1, Efg1, Sfl1, Sfl2, Ndt80, Mcm1), biofilm formation (Rob1), white-opaque switching (Wor1, Efg1), cell wall damage response (Cas5, Sko1), stress response (Cta8, Cwt1, Skn7, Sko1) and metabolism (Tye7, Ron1), whose target genes were enriched in the *sth1* transcriptome. Taken together, the integrated analysis of the transcriptome and proteome of *C*. *albicans sth1* mutant implicates CaRSC in the regulation of species- and niche-specific behaviors of the pathogen in addition to fundamental growth-related processes.

### Deletion of accessory RSC subunits affects filamentous growth and stress response in *C*. *albicans*

**Fig 4:**
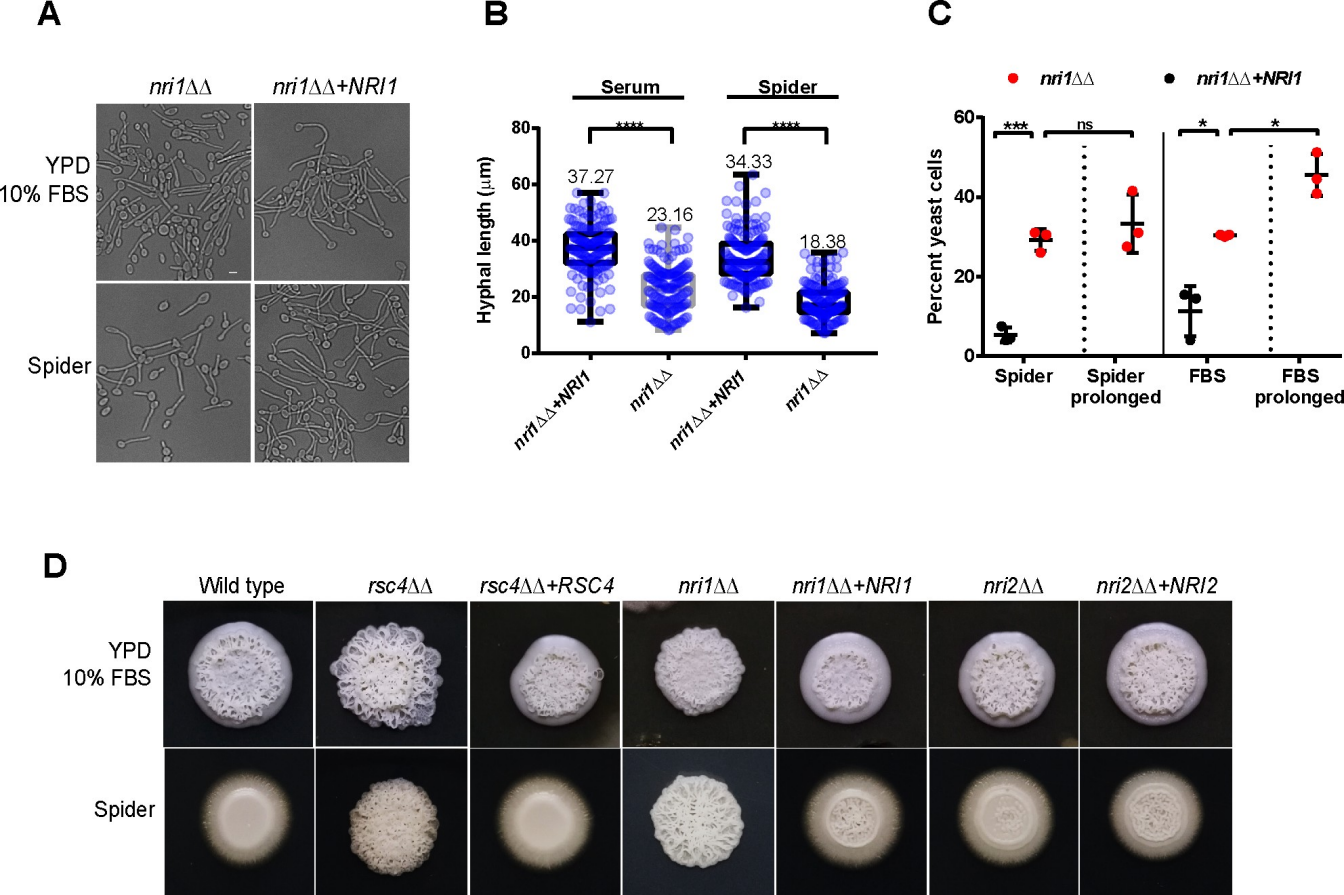
Filamentous growth and stress response of *rsc* mutants in *C*. *albicans*. **A.** Cellular morphology of *nri1ΔΔ* (SGC2047) and re-integrant control (SGC2049) induced to form hyphae in the indicated liquid media. Images were acquired after 90 minutes of induction at 37°C (bar-5 μm). **B.** Hyphal lengths of *nri1ΔΔ* and re-integrant control in the indicated media as described in A. Values from 150 cells per strain were analyzed from 3 independent experiments. Mean hyphal length for each condition is also given. Statistical analysis was performed using one-way ANOVA followed by Tukey’s multiple comparison test (****p<0.0001 in both cases). Raw data underlying graph given in S7 Table. **C.** Percentage of yeast form cells in *nri1ΔΔ* (SGC2047) and re-integrant cells (SGC2049) induced to form hyphae in the indicated liquid media for 90 and 150 (prolonged) minutes. Each data point represents mean value of 200 cells. Test of significance was performed using unpaired *t* test with Welch’s correction (p<0.05 in all cases). Error bar denotes standard deviation. Raw data underlying graph given in S7 Table. **D.** Colony morphology of the indicated strains (WT:SGC2024; *rsc4ΔΔ*:SGC2022; *rsc4ΔΔ*+*RSC4*:SGC2023; *nri1ΔΔ*:SGC2047; *nri1ΔΔ*+*NRI1*:SGC2049; *nri2ΔΔ*:SGC2032; *nri2ΔΔ*+*NRI2*:SGC2036) on the indicated hyphae-inducing solid media at 37°C. Colonies were photographed after 5 days of growth.

*STH1* is essential for viability, making phenotypic analysis of the mutant for stress response or morphogenesis hard to perform. Therefore, to correlate the transcriptomic and proteomic changes observed by loss of RSC function after conditional inactivation of *STH1* with phenotypes, we investigated the phenotypic consequences of RSC inactivation in *C*. *albicans* focusing on the non-essential Rsc4 subunit, as well as the novel CTG-clade specific Nri1 and Nri2 subunits identified in this study (Fig 1E and 1F). An important determinant of *C*. *albicans* virulence is the ability to switch from yeast to hyphal forms [35, 36]. In response to 10% FBS and in Spider media, which both induce hyphal morphogenesis, we determined the ability of *rsc4*ΔΔ, *nri1*ΔΔ and *nri2*ΔΔ mutants to form hyphae after 90 minutes of induction. While *rsc4*ΔΔ and *nri2*ΔΔ cells formed hyphae similar to wild type in liquid media (S6 Fig), *nri1*ΔΔ cells seemed to form fewer and shorter hyphae in both FBS and Spider media (Fig 4A). We observed that the hyphal lengths of *nri1*ΔΔ were significantly shorter than those of *nri1*ΔΔ+*NRI1* rescue strain in both Spider and FBS media (Fig 4B). Moreover, around 30% of *nri1*ΔΔ cells did not form germ tubes and remained in yeast form in these media as opposed to 5–10% cells of *nri1*ΔΔ+*NRI1* (Fig 4C). Although this phenotype could stem from the inherent slow growth of *nri1*ΔΔ (Fig 1E), even after prolonged (150 min) hyphal induction, 33% and 45% cells in yeast form were observed in Spider and FBS media respectively (Fig 4C). A further significant increase in yeast form population upon prolonged induction of *nri1*ΔΔ in FBS media might be explained by the faster mitotic divisions of yeast cells in FBS (in YPD) compared to Spider medium which contains the non-preferred carbon source mannitol instead of glucose. Interestingly, on FBS-containing plates observed after 5 days, *rsc4*ΔΔ formed hyper-wrinkled colonies and this phenotype was shared with *nri1*ΔΔ to a lesser extent (compare peripheral region of mutant and control colonies, Fig 4D top panel). Similarly, wrinkled colony morphologies were observed on Spider plates for *rsc4*ΔΔ and *nri1*ΔΔ, but both these mutants were defective in lateral hyphal growth (Fig 4D bottom panel). Phenotype on plate was largely unaffected in *nri2*ΔΔ mutant as the colony morphologies resembled that of control strains in both media.

**Fig 5:**
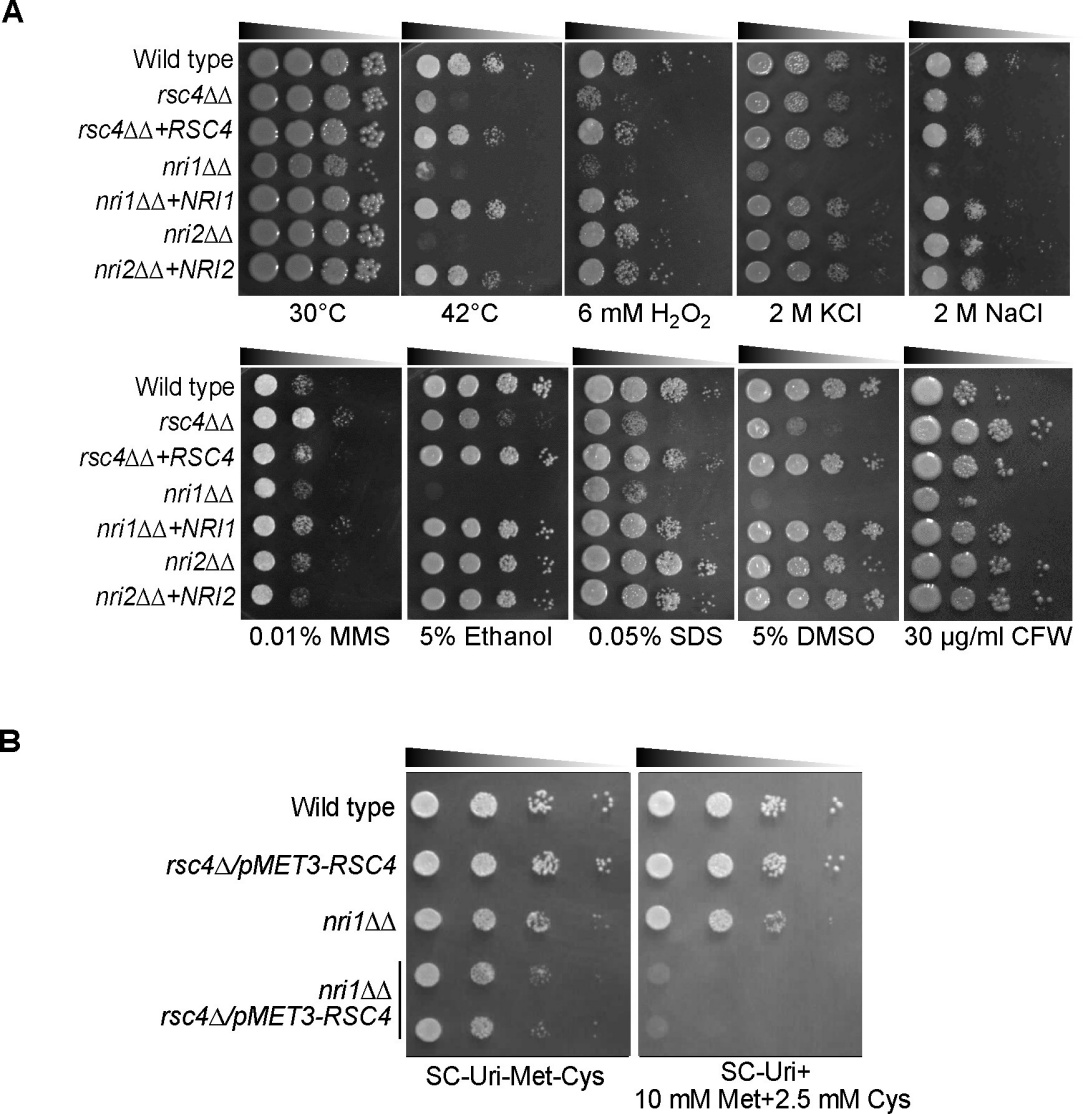
Functional links between *RSC4* and *NRI1* revealed through phenotypic analysis and genetic interaction. **A.** Growth phenotypes of *rsc4ΔΔ*, *nri1ΔΔ* and *nri2ΔΔ* mutants along with the corresponding re-integrants and wild type (WT:SGC2024; *rsc4ΔΔ*:SGC2022; *rsc4ΔΔ*+*RSC4*:SGC2023; *nri1ΔΔ*:SGC2047; *nri1ΔΔ*+*NRI1*:SGC2049; *nri2ΔΔ*:SGC2032; *nri2ΔΔ*+*NRI2*:SGC2036) under various *in vitro* stress conditions. Serial dilutions of cells were spotted on YPD plates with the indicated stressors and incubated at 30°C, except where mentioned otherwise, and photographed after 2–3 days. **B.** Genetic interaction between *RSC4* and *NRI1*. Serial dilutions of the indicated strains (WT:SGC2006; *MET3-RSC4*:SGC2053; *nri1ΔΔ*:SGC2047; *nri1ΔΔ MET3-RSC4*:SGC2084) were spotted on growth permissive (SC-Uri-Met-Cys) and repressive (SC-Uri+10 mM Met+2.5 mM Cys) plates and incubated at 30°C for 2 days before they were photographed.

We extended our phenotypic analysis to assess the impact of exogenous stress factors on the growth of *rsc4*ΔΔ, *nri1*ΔΔ and *nri2*ΔΔ mutants (Fig 5A). Among these mutants, *nri1*ΔΔ exhibited strong growth defects in response to physiologically relevant factors such as thermal (42°C), oxidative (H_2_O_2_), osmotic (KCl and NaCl), membrane (DMSO and ethanol) and cell wall (Calcofluor white and SDS) stresses. It is notable that the *rsc4*ΔΔ mutant, albeit with a lesser extent, shared most of the *nri1*ΔΔ phenotypes, except for a marginal resistance to MMS (DNA damaging agent) and calcofluor white (cell wall damaging agent). These phenotypic similarities led us to probe if *RSC4* and *NRI1* interact at the genetic level. Since we failed to isolate viable double knockouts of *RSC4* and *NRI1*, a homozygous mutant of *NRI1* was generated in the *pMET3-RSC4* conditional mutant. We observed that *rsc4 nri1* failed to grow under growth repressive conditions (Fig 5B), indicating a synthetic lethal interaction between *RSC4* and *NRI1*. This further potentiates Nri1 as a component of RSC complex. On the other hand, thermal stress was the only factor in our screening that conferred sensitivity to *nri2*ΔΔ cells and thus, the only condition that affected the growth of all three mutants. Collectively, our phenotypic data indicate that *RSC4* and *NRI1* are required for proper response of *C*. *albicans* to *in vitro* filamentation and stress-inducing cues (Fig 4A–4D and Fig 5A) and their combined loss is lethal for cell survival (Fig 5B).

### Loss of Rsc4 attenuates *C*. *albicans* virulence in a mammalian host

**Fig 6:**
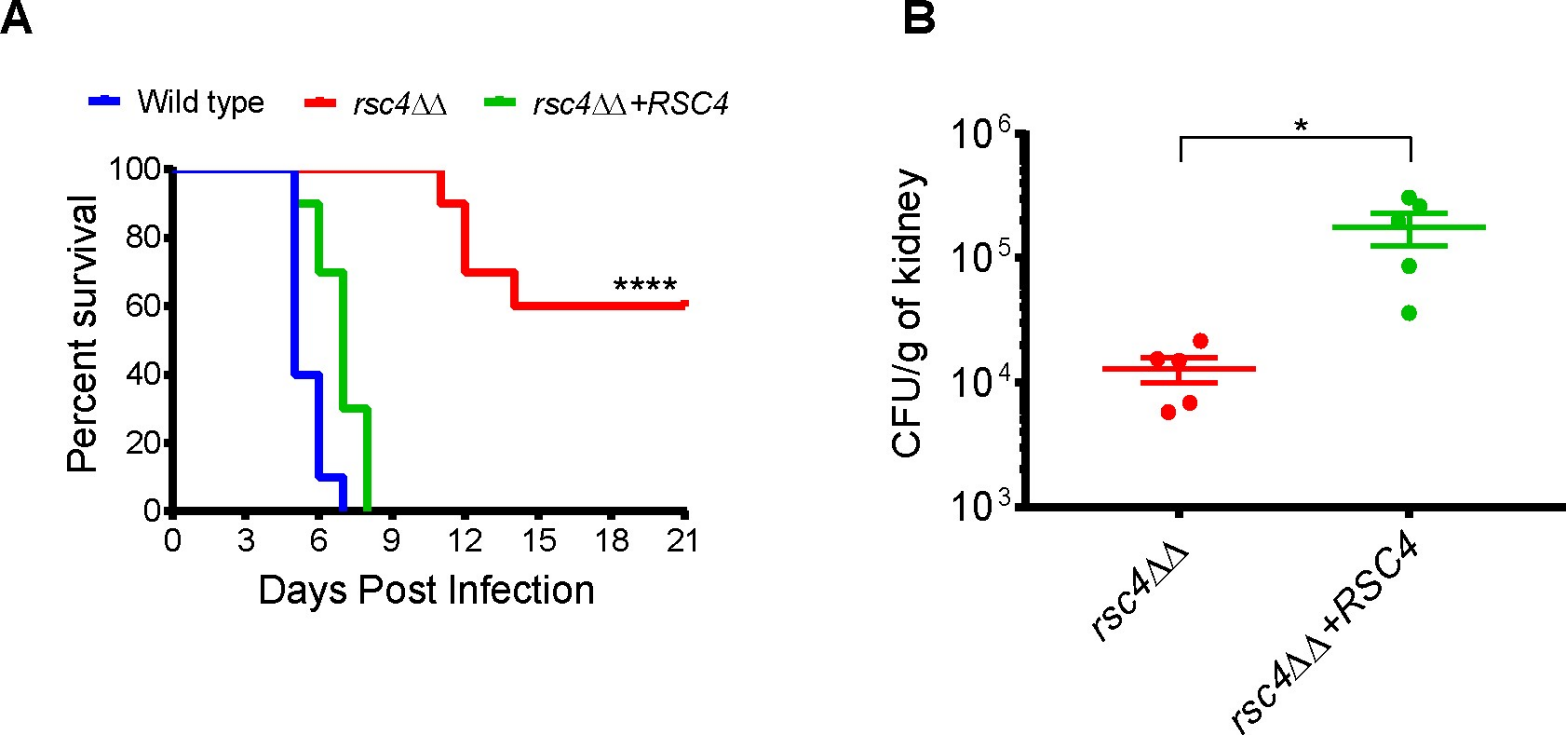
Rsc4 contributes to virulence of *C*. *albicans* in a mice systemic infection model. **A.** Survival kinetics of mice intravenously injected with 3X10^5^ cells of wild type (SGC2024), *rsc4ΔΔ* (SGC2022) and re-integrant strains (SGC2023). Statistical analysis was performed using Mantel-Cox test (****p<0.0001; 10 mice per strain). Raw data underlying graph given in S7 Table. **B.** Kidney fungal burden estimated from mice at 3 days post infection with the indicated *C*. *albicans* strains (5 mice/strain). Each data point represents the CFU/g of kidney tissue from individual mice of each category. Test of significance was performed using unpaired *t* test with Welch’s correction (*p<0.05). We have shown the complemented strain rather than the wild type as the positive control (see Materials and methods for details). Raw data underlying graph given in S7 Table.

The ability to sense and adapt to complex microenvironmental conditions is critical for a pathogen’s fitness inside the host and mutants with defects in proper adaptive responses show impaired survival [37]. Given the complex phenotypes of RSC mutants (Fig 4A–4D and Fig 5A), we assessed the pathogenic potential of *rsc4*ΔΔ using *in vivo* (mouse) and *ex vivo* (primary immune cells) models. First, we challenged female BALB/c mice with *rsc4*ΔΔ mutant and the re-integrant control in a systemic infection mouse model. All mice infected with the wild type or re-integrant (rescue strain) strain were killed within 9 days of infection, while 60% of the mutant-infected mice survived during the entire observation period (Fig 6A), and the kidney fungal burden of *rsc4*ΔΔ-infected mice was significantly lower compared to the control strain (P<0.05, Fig 6B).

We next addressed if the reduced virulence of *rsc4*ΔΔ was due to increased susceptibility to phagocyte killing. For this purpose, we challenged human neutrophils with wild type, *rsc4*ΔΔ and *rsc4*ΔΔ*+RSC4* and estimated the intracellular killing of fungal cells (multiplicity of infection: 1:1). As represented in S7A Fig, *rsc4*ΔΔ showed only a marginal increase in percent death compared to wild type and re-integrant cells. We also infected mouse primary bone marrow-derived macrophages with these strains and monitored the kinetics of macrophage death, which is a read-out of the ability of *C*. *albicans* to form robust hyphae to trigger the initial cell death and escape [38], followed by robust fungal replication that triggers glucose starvation in macrophages and the rapid death seen later in infection [39] (S7B Fig). However, we did not observe any differences in the death rate of macrophages infected with *rsc4*ΔΔ relative to those infected with wild type or re-integrant cells. Taken together, our data reveals that the deletion of *RSC4* attenuates *C*. *albicans* virulence and immune clearance of *rsc4*ΔΔ is less likely to contribute to this phenotype.

## Discussion

RSC is a multiprotein chromatin remodeling complex with defined roles in regulating the global transcriptional status of an eukaryotic cell. The composition of this conserved complex is known to vary in the model yeasts in terms of loss or gain of specific accessory subunits and their essentiality [16]. Importantly, such variations contribute towards the species-specific functional divergence of the complex, which necessitates its analysis across diverse lineages. Our biochemical and genetic studies of the *C*. *albicans* RSC complex highlight several aspects along these lines. First, the 13-member CaRSC represents a more concise version of its *S*. *cerevisiae* counterpart, which is comprised of 17 subunits (Fig 1D, 1G). This is because many of the bonafide ScRSC subunits (Rsc3, Rsc30, Htl1, Ldb7) are not encoded by the *C*. *albicans* genome, although we cannot exclude the possibility of other proteins serving as functional homologs of these subunits in this organism. Furthermore, only single isoforms of the paralogous subunits ScRsc1-Rsc2 were identified in the CaRSC. One possibility is that these additional *S*. *cerevisiae*-specific subunits might have originated as a result of the whole genome duplication event (WGD) that took place approximately 100 million years ago [40]. In support of this premise, orthologs of Rsc3-Rsc30 [41] and Htl1 and Ldb7 (CGD) have been annotated in another WGD-descendent *Candida glabrata*, a fungal pathogen found in the same habitat as the non-WGD *C*. *albicans*. Our Sth1 AP-MS data also suggests that the CaRSC lacks Rtt102 (Fig 1B), a subunit shared by RSC and SWI/SNF complexes in *S*. *cerevisiae*. The ScRtt102 assists in forming a stable association of Arp7-Arp9 but is not essential for their recruitment into the complex [42, 43]. Interestingly, Rtt102 was also not identified in the CaSWI/SNF complex, although the CaArps were detected [12]. It would be intriguing to see if the CaArp7-Arp9 might associate and function in the SWI/SNF complexes in an Rtt102-independent manner. Second, we report here the identification of two novel CTG clade-specific RSC subunits Nri1 and Nri2 in *C*. *albicans* (Fig 1B and 1C and S2 Table). Sequence analysis indicates that homologs of these proteins are present only in the CTG clade of fungi, which are characterized by a codon bias in translating the CUG codon to serine instead of leucine. It is notable that these proteins were not identified in the CaSWI/SNF [12], suggesting that these interactors are RSC-specific. Finally, several bonafide CaRSC subunits deviate in their requirement for viability when compared to the model yeasts (Fig 1F). The bromodomain-containing subunits that we selected (Rsc1/Rsc2, Rsc4 and Rsc58; Fig 1F) are all essential in *S*. *cerevisiae* but not in *S*. *pombe* [16]. *C*. *albicans* is different to both of these model yeasts, as our data shows that Rsc4 is non-essential, while Rsc1 and Rsc58 are essential in this pathogen (Fig 1F).

Conditional knockout of *CaSTH1* led to broad changes in gene expression. Some of the major changes observed include repression of ribosomal protein genes in addition to repression of genes associated with energy metabolism and mitochondrial functions (Fig 3A). In contrast, protein catabolism-related transcripts and proteins were induced in *sth1* (Fig 3A and 3B). Conceivably, alterations in ribosomal and proteasomal functions in *sth1* could increase the disparity between the transcriptome and proteome profiles. Despite this, we observed a good correlation between the transcripts and proteins that are mis-regulated in *sth1* (Fig 2B). We also identified that the differentially expressed genes in *sth1* contain known targets of a number of TFs (Nrg1, Efg1, Wor1, Ndt80, Cas5, Sko1, Tye7) which are relevant to the pathogenic behavior of *C*. *albicans* (S5 Fig), suggesting that RSC functions with these TFs in the regulation of gene expression. In addition, some of these TFs (Nrg1, Efg1 and Hap43) were found to be differentially expressed in the *sth1* transcriptome (S5 Fig; S3 Table). Given the role of ScSth1 in the transcriptional activation of Pol I, Pol II and Pol III transcribed genes [24], we were surprised to observe a roughly equal proportion of upregulated and downregulated genes in the *C*. *albicans sth1* mutant. Notably, many of these induced genes/proteins are required for responses to specific biotic or abiotic stresses and not for normal growth. Whether the altered regulation of genes/proteins is a direct consequence of loss of RSC function, or caused indirectly due to a global stress response which would be expected upon loss of an essential chromatin regulator, remains to be understood. It should be noted that RSC has been shown to repress transcription through nucleosome maintenance in the promoter of a highly inducible gene [44]. Besides, a cofactor-dependent recruitment of RSC to the histone locus (*HTA1-HTB1*) was associated with histone gene repression outside the S phase of cell cycle [45]. These findings imply that RSC might also function in suppressing transcription to prevent untimely gene expression and that the overall transcriptional outcome of RSC activity could be dependent on the locus or cellular context.

Using the non-essential components of the CaRSC identified in this study (Rsc4, Nri1 and Nri2), we demonstrated the role of RSC in hyphal growth and stress responses of *C*. *albicans*. Except for Nri1, these proteins were found dispensable for hypha formation in liquid cultures (Fig 4A–4C and S6 Fig). However, the formation of lateral filaments was impaired in *rsc4ΔΔ* and *nri1ΔΔ* on Spider containing plates and unlike wild type, the colonies of these cells appeared more wrinkled on FBS plates (Fig 4D). Such conditional defects in morphogenesis on the solid media reflect the impact of niche-specific conditions more than the primary inducing cue/s (e.g. serum and temperature). Unlike in liquid cultures which assay the response of *Candida* to the primary inducing cue/s, phenotype on solid media can be influenced by additional factors such as colony microenvironment, quorum sensing, nutrient availability and oxygen tension [4]. Previous work showing that mutants commonly have distinct filamentation phenotypes in liquid versus solid medium [46] fully supports our data with the *C*. *albicans* RSC mutants. It has also been shown that the gene expression programs differ between liquid and solid hyphae-induction conditions [46]. It will be interesting to determine how RSC controls hyphal gene expression in liquid and solid conditions. Stress response assays, on the other hand, revealed broad phenotypic defects in *nri1ΔΔ* and to a lesser extent, in *rsc4ΔΔ* (Fig 5A). Interestingly many of the *nri1*ΔΔ phenotypes closely mirrored a bona fide RSC mutant *rsc4*ΔΔ (Fig 5A), an observation typical of proteins occupying the same complex. Besides, we showed that the combined loss of *RSC4* and *NRI1* is detrimental to cell viability (Fig 5B). It appears from these results that the two subunits might positively cooperate to regulate the expression of gene/s involved in essential function/s. However, the composition of CaRSC is unaffected in *rsc4*ΔΔ mutant (S2 Fig). In this context, we propose that loss of both Rsc4 and Nri1 might affect the organizational integrity of the CaRSC and/or perturb the targeting of CaRSC to essential gene/s. In contrast, the functions of Nri2 largely remain enigmatic, with a growth defect at 42°C being the only phenotype found. It was surprising to note that despite these phenotypic defects, particularly sensitivity to oxidative stress, we observed little effects on the interaction of *rsc4ΔΔ* strain with phagocytes (S7A and S7B Fig). One possibility is that the level of H_2_O_2_ exposure we tested (Fig 5A) might have exceeded the physiologically relevant threshold of ROS (Reactive Oxygen Species) in the phagocytes. Alternatively, the pleiotropic effects of *RSC4* mutation might explain the discrepancy between the two phenotypes as a similar scenario has been reported for the H_2_O_2_-sensitive thioredoxin (*TRX1*) mutant in *C*. *albicans* [47]. Nevertheless, we demonstrated that knockout of *RSC4* compromises the virulence of *C*. *albicans* in a murine systemic infection model (Fig 6A and 6B), showing that this subunit is required for full fitness of *C*. *albicans* in the host.

In summary, we showed that the chromatin remodeling complex RSC is compositionally and genetically distinct in *C*. *albicans* compared to model yeasts *S*. *cerevisiae* and *S*. *pombe*, and conducts important functions in the survival, adaptive fitness and virulence of the pathogen. The increasing concern with *C*. *albicans* is that with the emergence of drug-resistant clinical isolates, existing anti-*Candida* drugs are proving to be less efficacious (reviewed in [48]). Consequently, in line with the demand to expand the toolbox of molecular drug targets, inhibition of pathogenic attributes such as the dimorphic switch are extensively focused. However, species such as *C*. *glabrata* and *C*. *auris* do not respond to cues that typically trigger filamentation in *C*. *albicans* [49, 50], yet contribute to high mortality rates [51, 52]. This emphasizes the need to focus on molecular aspects that are conserved among fungal pathogens and that affect not just virulence but also survival of the pathogen. Our findings make the CaRSC attractive in this context. Despite the complex being conserved across the fungal pathogen and the human host, small molecule inhibitors selectively targeting distinct catalytic residues could serve as potent antifungal agents as demonstrated elsewhere [53]. Alternatively, targeting *Candida*-specific subunits (Nri1 and Nri2) of the complex presents yet another possibility to explore in the future. Nevertheless, unraveling the spectrum of CaRSC functions and its roles in the biology of *C*. *albicans* is paramount to set the groundwork for such attempts.

## Materials and methods

### Strains, media and culture conditions

The *C*. *albicans* strains, plasmids harboring cassettes used for *Candida* strain construction and oligonucleotides used in this study are described in S6 Table. All conditional mutant strains used in this study were derived from SN148, whereas SN152 was used to create *rsc4ΔΔ*, *nri1ΔΔ*, *nri2ΔΔ*, and their corresponding re-integrants. The re-integrants were constructed by cloning full-length ORFs along with 5’ and 3’ UTRs in the pSN complementation vector [54], which integrates at *LEU2* locus. Each of the null mutants was marker-matched to their respective re-integrants by integrating the empty pSN vector. Growth assays to check the functionality of epitope-tagged proteins used in this study is given in S9 Fig.

General conditions for *C*. *albicans* growth were YPD (1% yeast extract, 2% peptone and 2% glucose) supplemented with 80 μg/ml uridine at 30°C. For selection of transformants, YPD + 200 μg/ml nourseothricin (Werner BioAgents) or synthetic media lacking the appropriate amino acid/s was used.

Hyphal induction in liquid media was performed by diluting overnight cultures to 0.5 OD in pre-warmed YPD+10% FBS (fetal bovine serum) or Spider (1% nutrient broth, 1% D-mannitol and 0.2% dibasic potassium phosphate, pH 7.2) media and incubated at 37°C for the time periods indicated in figures. Imaging was performed at 40X magnification using AxioObserver Z1 (Zeiss). Hyphal lengths were measured using the ‘segmented line’ tool of ImageJ (https://imagej.nih.gov/ij/) from the edge of yeast cell to the hyphae tip. Pixel-based measurements were converted to micrometers using microscope scale bar settings. For plate-based filamentation assays, indicated strains were spread plated on pre-warmed YPD+10% serum or Spider or RPMI media and incubated at 37°C for 5–7 days before the colonies were photographed.

Sensitivity to stress conditions was assessed by spotting ten-fold dilutions of mutant and control strains on plates containing various drugs and chemicals. The plates were photographed after 2–3 days of incubation at 30°C, or 37°C or 42°C as indicated in the Figures.

### Affinity purification followed by mass spectrometry (AP-MS)

Overnight culture of wild-type and tagged strains were adjusted to 0.2 OD in YPD. Approximately 2 X 10^9^ mid-log-phase cells were harvested and proceeded for single step affinity purification. The protocol for immuno-purification was adapted from Byrum et al [55] with slight modifications. Briefly, frozen cell pellets were resuspended in lysis buffer (20 mM HEPES, pH 7.5, 2 mM magnesium chloride, 250 mM sodium chloride, 0.1% Triton X-100 and freshly prepared protease inhibitor cocktail (Roche)) and lysed by bead-beating for 10 cycles of 30 seconds with intermittent cooling on ice. Lysates were precleared using Protein G-conjugated Dynabeads (Invitrogen, 10003D). Anti-Myc antibody (9E10, mouse monoclonal) was added to the lysate at a final concentration of 10 μg/ml and incubated at 4°C with rotation. After 2 hours, Protein G-coupled Dynabeads were added and further incubated for 2 hours at 4°C. The beads were once washed using lysis buffer followed by four washes with wash buffer (20 mM HEPES, pH 7.5, 2 mM magnesium chloride, 200 mM sodium chloride) at 4°C. Acidic elution with 0.2 M glycine pH 2.5 was performed at room temperature and the eluate was neutralized with 1 M Tris pH 8. The eluted proteins were reduced with 10 mM tris (2-carboxyethyl) phosphine hydrochloride (TCEP) and cysteine-alkylated with 40 mM chloroacetamide (CAA) before overnight digestion with sequencing grade trypsin (Trypsin Gold, Promega). The mixture was acidified with formic acid to inactivate trypsin and desalted using ziptips packed with C18 resin (Agilent Technologies). Trypsin-digested samples were dried in a SpeedVac and reconstituted in Mass Spectrometry Sample Loading Buffer (0.1% formic acid spiked with 10 pmole iRT (indexed retention time) peptides, Biognosys) for LC-MS/MS analysis.

The peptides were separated on Dionex UltiMate 3000 RSLC Ultra High Performance Liquid Chromatography system (Thermo Fischer Scientific) by injecting 6 μl sample onto an Acclaim PepMap RSLC analytical column (75 μm wide, 50 cm long, nanoViper C18 with 2 μm particle size and 100 Å pore size (Thermo Fischer Scientific)) using a 158 min in-house gradient protocol. Mass spectra were acquired in a data-dependent mode on a QExactive Plus Orbitrap mass spectrometer (Thermo Fisher Scientific) connected in-line to the LC system. Up to twelve most abundant precursor ions were isolated from a survey scan of 375–1600 *m*/*z* at 70,000 resolution for fragmentation in an HCD (High energy Collisional Dissociation) cell. MS2 scans were performed at a resolution of 17,500 with a scan range of 200–2000 *m/z* and dynamic exclusion of 15 s to minimize repeated selection of precursor ions for fragmentation.

### AP-MS data analysis

#### Identification of Sth1 interactors

The acquired raw files were analyzed with MaxQuant (1.6.0.16) using the built-in Andromeda search engine to identify and quantify proteins. Searches were performed against the *C*. *albicans* proteome database downloaded from UniProt (May 2017 with 6040 entries) and appended with the iRT peptide sequences. The following settings have been used: trypsin for enzyme specificity with a maximum of 2 missed cleavages, cysteine carbamidomethylation as fixed modification, methionine oxidation and protein-N terminal acetylation as variable modifications, minimum peptide length set to 7, and maximum peptide mass to 6000 Da. The peptides and proteins were identified with a false discovery rate of 0.01. Label-free quantification of proteins was performed using razor peptides considering at least 2 peptides for pair-wise comparison of a protein between two samples. Perseus (1.6.0.7) was used to statistically analyze the MaxQuant output data based on the ProteinGroups text file using the following workflow: proteins identified as reversed, potential contaminants or identified by only site have been removed. The LFQ intensities were log_2_ transformed and imputed to replace missing values from normal distribution. We utilized the volcano plot function which uses a two-sample t-test to estimate if the mean LFQ intensities of the two groups were significantly different. The threshold for identifying significant interactors is represented by the parabola, defined by a combination of permutation-based FDR and S0, representing a minimum fold change (when S0 is set to 0, FDR alone determines the difference between the two groups).

#### Identification of Nri1 and Nri2 interactors

The acquired raw files were searched against the *C*. *albicans* proteome database (as mentioned above) using Byonic (v3.1.0, Protein Metrics) with the following parameters: digestion by C-terminal cutter (trypsin) with maximum 2 missed cleavages per peptide, carbamidomethylation (C) as fixed and, oxidation (M) and protein N-terminal acetylation as variable modifications. The precursor and fragment mass tolerance were set to 10 and 20 ppm respectively, with HCD as the fragmentation type. Decoy-based false discovery rate of protein identification was set to 1%. Only proteins that have been exclusively identified in the Nri1 and Nri2 pulldown samples, but not in the controls (untagged and minus antibody samples), were deemed significant (S2 Table). For each condition, biological duplicates were analyzed.

### Co-immunoprecipitation

Around 8 X 10^8^ cells were harvested from wild-type and tagged strains. Whole cell protein extraction and immuno-purification were performed as described for AP-MS with slight changes. Polyclonal rabbit anti-Myc antibodies (ab9106, Abcam) were used for immunoprecipitation of bait (Sth1-Myc) and beads were eluted by boiling in SDS-PAGE loading buffer. Proteins were separated on an 8% SDS polyacrylamide gel and transferred to nitrocellulose membrane for immunoblotting.

### Western blotting

Standard procedures were followed for western blotting. Sth1-Myc was detected using monoclonal mouse anti-Myc antibodies (9E10, Roche) and HA-tagged proteins with monoclonal rat anti-HA antibodies (3F10, Roche). The loading controls actin and tubulin were detected with monoclonal mouse anti-actin (Millipore) and monoclonal rat anti-tubulin (Bio-Rad). Appropriate secondary antibodies conjugated to HRP (Jackson ImmunoResearch and Genei) were used for developing the blots.

### Microscopic analysis of mitochondrial morphology

Harvested cells were washed and stained with 0.1 μM MitoTracker Red CMXRos (Thermo Fischer Scientific) in YPD for 30 minutes at 30°C. Cells were washed thrice with phosphate buffered saline (PBS) post-staining and imaged with EVOS FL Auto Imaging system.

### RNA-Seq

Sth1-dependent transcriptome changes were monitored by analyzing RNA extracted from *sth1*Δ*/pTetO-STH1-MYC* and *sth1*Δ*/STH1-MYC* cells. Overnight cultures were adjusted to an initial OD of 0.05 in YPD and grown for 2 hours before adding 20 μg/ml doxycycline. Depletion was performed for 7 hours to ensure Sth1 depletion without compromising cell viability (S2D Fig) along with minus dox control. Three biological replicates per condition were analyzed. Total RNA was extracted from frozen cell pellets using hot phenol method and assessed for integrity on 2100 Bioanalyzer (Agilent). Four micrograms of RNA were used to prepare poly-A RNA-Seq libraries using TruSeq Stranded mRNA Sample prep (Illumina Protocol 15031047). The mean size of the libraries ranged from 340–367 bp. Single-end short-read sequencing (80 bp) of the libraries on HiSeq3000 generated around 30 million reads per sample. The in-house RNAsik pipeline [56] mapped around 90% of all reads to the *C*. *albicans* reference genome downloaded from the Candida Genome Database (SC5314-assembly 22). The raw count files from RNAsik were analyzed by Degust [57], which relies on limma-voom [58] to identify differentially expressed genes that are statistically significant. Variations in library sizes across samples were accounted for using CPM (Counts Per Million) normalization, whereas the TMM (Trimmed Mean of M values) method [59] was applied to normalize for bias in RNA composition. Genes with a minimum of 1 CPM in at least four samples were considered for the analysis. False discovery rates of the log_2_ transformed fold-change values were estimated by calculating p-values adjusted for multiple testing. The entire RNA-Seq dataset can be accessed using the link - http://degust.erc.monash.edu/degust/compare.html?code=b353d0c2039cd97051bcd5651bc0db19#/

### Data independent acquisition-mass spectrometry (DIA-MS)

For DIA-MS, samples were harvested as described for RNA-Seq analysis. Cells were lysed with Tris (pH 8.1) containing 1% (w/v) SDC (sodium deoxycholate) and the lysate was boiled, sonicated and clarified by centrifugation at 4000 rpm for 5 minutes. Proteins were reduced, alkylated and trypsin-digested, and SDC was removed by phase separation with 100% water-saturated ethyl acetate. Desalting and sample processing for mass spectrometric analysis was done as mentioned for AP-MS.

To generate a comprehensive spectral library of *C*. *albicans* proteins, 700 μg of protein extract was trypsin-digested and the peptides were separated using off-line high-pH reversed-phase fractionation method described previously [60]. Forty fractions were collected and equidistantly pooled into 6 samples.

Mass spectra were acquired on Orbitrap Fusion Tribrid mass spectrometer (Thermo Scientific) connected in-line to the UHPLC system described above. For DIA analysis, each sample was injected twice with MS1 scan parameters as follows: 120000 resolution, scan range 375–1575 *m/z*, maximum injection time 54 ms and automatic gain control (AGC) 10^7^. Parameters for orbitrap MS2 scan were set to quadrupole isolation of width 12 *m/z*, 30000 resolution, scan range 200–2000 *m/z*, maximum injection time 54 ms and AGC 10^7^. All samples to create the spectral library were acquired in the data-dependent mode with identical MS1 scan settings. MS2 scans were acquired with quadrupole isolation of width 1.4 *m/z*, resolution 30000, maximum injection time 118 ms, AGC 400000 and, dynamically excluded for 15 s.

### Analysis of DIA-MS data

The spectral library was generated in Spectronaut Pulsar (Biognosys) after all DDA files were analyzed with Proteome Discoverer (2.2). The DIA raw files were converted to the HTRMS format and analyzed with Spectronaut using the BGS factory default settings. A q-value threshold of 1% was assigned for precursor and protein identifications and, quantification was performed at the MS2 level based on area under the XIC peaks. Differential abundance of proteins between conditions were evaluated using Student’s t-tests and candidates passing a q-value threshold of 0.05 were exported for further analysis.

### Analysis of *sth1* gene expression profiles

Gene ontology (GO) analysis was performed on differentially expressed genes and proteins filtered by 1.5 fold change and 0.05 FDR, using the GO Slim mapper tool from the CGD (http://www.candidagenome.org/cgi-bin/GO/goTermMapper).

GSEA was performed using standard parameters [9]. Gene lists were filtered as mentioned for GO analysis above. For analysis of *sth1* proteome dataset, fold change cutoff was not applied. Enrichment maps were created in Cytoscape v3.5.1 with the following settings: 0.005 p-value, 0.1 FDR and overlap threshold 0.5.

TF analysis of *sth1* transcriptome (1.5-fold change, 0.05 FDR) was done using PathoYeastract [61] on June 8 2019, which contains a curation of transcription factors-target associations gathered from DNA-binding and expression-based experimental evidence in *C*. *albicans*. The “Rank by TF” option of PathoYeastract accepts a user-defined list of genes (here the *sth1* transcriptome) and predicts the TFs regulating these genes by performing an enrichment analysis. For every predicted TF, the probability of overrepresentation of TF targets in the input list by chance is denoted by a p-value based on a hypergeometric distribution. The most relevant TFs were identified by filtering the list using p-value 0 and targets comprising a minimum of 5% of the input genes; and represented in the form of an interaction network generated using Cytoscape.

### Immune cell interaction assays

Neutrophils were isolated from the blood of human donors and infected with wild type, *rsc4*ΔΔ and *rsc4*ΔΔ*+RSC4* cells at a MOI (multiplicity of infection) of 1:1 (*Candida*:Neutrophil). *Candida* cells without neutrophils served as control. After 1 hour of incubation at 37°C, the neutrophils were lysed using cold water to release the internalized yeast cells. Serial dilutions of the suspension were prepared and plated on YPD plates for enumeration of colony forming units. Percentage killing of *Candida* by neutrophils was determined as follows–(CFUs from *Candida* with neutrophils / CFUs from *Candida* alone) X 100.

Macrophage assay was performed as described [62]. Briefly, macrophages derived from mouse bone marrow were infected with *Candida* at 1:1 MOI. Macrophage death was assessed over a period of 24 hours by monitoring DRAQ7 fluorescence using live cell microscopy. Data from 2 biological replicates are presented.

### Mice model infection and kidney fungal burden assays

Mice experiments were performed in compliance with the guidelines from the Monash University Animal Ethics Committee (approval number MARP-2015-170-TRAVEN). Mice infection experiments were performed as described [39]. Single colonies of wildtype, mutant and re-integrant strains were picked from YPD plates, washed with PBS and counted. 3 X 10^5^ cells of each strain were intravenously injected in 6–8 weeks old female BALB/c mice. Infected animals were scored for clinical signs of illness including weight loss over a period of 21 days. For estimation of kidney fungal burden, mice infected with *rsc4ΔΔ* and re-integrant cells were euthanized at 3 days post infection. The isolated kidneys were homogenized in PBS and plated on YPD containing ampicillin for enumeration of colony forming units. Statistical tests, as mentioned in legends (Fig 6A and 6B) were performed using GraphPad Prism 7.02.

For fungal burdens (Fig 6B), the *rsc4*ΔΔ mutant and *rsc4*ΔΔ+*RSC4* complemented strain were assayed alongside several other mutants and the parental wild type strain. The fungal burden data for the parental wild type was published alongside the other mutants elsewhere [63]. Importantly, the fungal burden for the *rsc4*ΔΔ+*RSC4* complemented strain was equivalent to the wild type strain (p = 0.994). Therefore, the data obtained from only the complemented strain is shown in Fig 6B as control.

## Supporting information

S1 Fig Deletion of *NRI1* affects growth of *C*. *albicans* under standard conditions. **A**. Growth of the indicated strains on YPD plates, photographed after 18 and 30 hours of incubation at 37°C. (Red dotted line indicates cropping within the same image). **B.** Growth curve of the indicated strains in YPD at 30°C. Absorbance at 600 nm was recorded every 90 minutes. Mean values with standard deviation from three independent experiments were plotted. Raw data underlying graph given in S7 Table.
(TIF)

S2 Fig Immunopurification of Sth1-Myc in *rsc4*ΔΔ of *C*. *albicans*. Volcano plot representation of significantly enriched proteins that copurified with Sth1-Myc in *rsc4*ΔΔ (SGC2011) compared to a no-tag control (SGC2006). Significant interactors identified are shown on the right side of the parabola as red dots, except for one potential interactor with low confidence score (Sfh1), highlighted on the left side. The grey boxes represent the potential background interactors. The two novel interactors are labelled in blue. The x-axis denotes the enrichment of the interaction in Sth1-Myc in *rsc4***ΔΔ** samples with respect to no-tag control, represented as a natural logarithm of normalized intensity values. The negative logarithm to the base 10 of the p-value, based on Student’s *t-*test, is given on the y-axis.
(TIF)

S3 Fig Depletion profile of Sth1 in *C*. *albicans*. **A**. Two independent clones of hemizygous and doxycycline-repressible conditional *sth1* mutants (*sth1*Δ*/STH1-*MYC:SGC2008; *sth1Δ/pTetO-STH1-MYC*:SGC2001) were spotted on YPD or YPD containing 20 μg/ml doxycycline (dox) plates. After 30 hours of growth at 30°C, the plates were photographed. **B.** Conditional *sth1* mutant (*sth1*Δ*/pTetO-STH1-MYC*) cells were treated with 20 μg/ml dox for the indicated time period and Sth1-Myc levels were monitored by immunoblotting using anti-myc antibody. Actin was used as the loading control and detected using anti-actin monoclonal antibody. **C.** Cellular morphology of hemizygous and conditional *sth1* mutants treated with 20 μg/ml dox for 7 and 24 hours respectively (bar-5 μm). **D.** Schematic showing the strategy to assess the viability of conditional *sth1* mutant treated with dox for 7 hours. *sth1*Δ*/pTetO-STH1-MYC* cells were initially cultured in the presence or absence of dox for 7 hours. Dox-treated cells were then split and re-passaged in media containing dox (RP+Dox) or lacking dox (RP-Dox). The untreated cells were re-passaged only in dox-lacking media (MT-Dox). The growth profiles of the cells in these cultures monitored in 96-well plates for 22 hours at 30°C with shaking at 200 rpm, are given below the schematic. Mean values with standard deviation are shown from 3 experiments. Raw data underlying graph given in S7 Table.
(TIF)

S4 Fig Altered mitochondrial morphology in Sth1-depleted *C*. *albicans* cells. Mitochondrial morphology assessed by MitoTracker staining of the indicated cells (*sth1*Δ*/STH1-*MYC:SGC2008; *sth1Δ/pTetO-STH1-MYC*:SGC2001) cultured with or without dox for 7 hours in YPD at 30°C with shaking at 200 rpm (bar—5 μm).
(TIF)

S5 Fig Transcription factor network analysis of *sth1* gene expression profile in *C*. *albicans*. The transcription factors (TFs) associated with *sth1* transcriptome profile identified from PathoYeastract database (analysis performed on 8 June 2019). Differentially expressed genes filtered by 1.5-fold change were analyzed and represented as an interaction network using Cytoscape. The circles other than Sth1, are shaded based on the percent of TF targets enriched in the dataset, as indicated by a color scale at the bottom. In addition, TFs mis-regulated in the *sth1* RNA-Seq dataset (1.5-fold change cutoff) are highlighted with a red border (if up-regulated) or green border (if down-regulated). A table mentioning the functions of the enriched TFs based on published literature is given on the right. Raw data underlying graph given in S7 Table.
(TIF)

S6 Fig Filamentous growth of *C*. *albicans* RSC mutants. Cellular morphology of wild type (SGC2024), *rsc4ΔΔ* (SGC2022) and *nri2ΔΔ* (SGC2032) induced to form hyphae in the indicated liquid media. Images were acquired after 90 minutes of induction at 37°C (bar-5 μm).
(TIF)

S7 Fig Interaction of *C*. *albicans rsc4ΔΔ* with immune cells. **A.** Intracellular killing of wild type (SGC2024), *rsc4ΔΔ* (SGC2022) and re-integrant (SGC2023) by human neutrophils (MOI 1) post 1 hour incubation, assessed by CFU counting. Percent death relative to no neutrophil control was plotted for each strain. Red and blue dots indicate percent values from two independent experiments and error bars denote standard deviation. Raw data underlying graph given in S7 Table. **B.** Kinetics of macrophage death in the presence of wild type (SGC2024), *rsc4ΔΔ* (SGC2022) and re-integrant (SGC2023) cells (MOI 1) assessed by live cell imaging for 24 hours. Mean values with SEM from two biological replicates were plotted, with at least 5000 macrophages surveyed for each strain. Raw data underlying graph given in S7 Table.
(TIF)

S8 Fig Uncropped western blot images of Sth1-Nri1 (C2_01370p-HA) and Sth1-Nri2 (C1_01800p-HA) co-immunoprecipitation from Fig 1C. Cell lysates from tagged and no tag control strains were immunoprecipitated with polyclonal anti-myc (ab9106) antibody. Sth1-Myc and Nri1-HA (C2_01370p-HA) or Nri2-HA (C1_01800p-HA) were detected by immunoblotting using monoclonal anti-Myc (9E10) and anti-HA (3F10) antibodies, respectively. Tubulin was used as the loading control. Two percent of input and 50% of eluate were loaded.
(TIF)

S9 Fig Growth analysis of strains with epitope-tagged proteins. Top panel shows the growth of wild type (SGC2006), *sth1*Δ*/pTetO-STH1-MYC* (SGC2001) and *STH1-MYC/STH1-MYC* (SGC2003) cells spotted on YPD or YPD containing 20 μg/ml dox plates. Bottom panel shows the growth of wild type (SGC2006), *MYC*-tagged *NRI1* (SGC2017) and *NRI2* (SGC2019), and their respective null mutants (*nri1ΔΔ*:SGC2047; *nri2ΔΔ*:SGC2032) at 30°C and 42°C. Images were taken after 2 days of incubation.
(TIF)

S1 Table AP-MS analysis of Sth1-Myc. (XLSX)

S2 Table AP-MS analysis of Nri1-Myc and Nri2-Myc. (XLSX)

S3 Table RNA-Seq data of conditional *sth1* mutant. (XLSX)

S4 Table DIA-MS data of conditional *sth1* mutant. (XLSX)

S5 Table GSEA analysis of *sth1* RNA-Seq and DIA-MS datasets. (XLSX)

S6 Table List of strains, plasmids and oligos used in the study. (XLSX)

S7 Table Raw data underlying Fig 2, Fig 4, Fig 6, S1 Fig, S3 Fig, S5 Fig and S7 Fig. (XLSX)
