# Supplementary figures and images for: The RSC (Remodels the Structure of Chromatin) complex of Candida albicans shows compositional divergence with distinct roles in regulating pathogenic traits
Source: PLoS Genet. 2020 Nov 5;16(11):e1009071. doi: 10.1371/journal.pgen.1009071 (PMC7671503; doi:10.1371/journal.pgen.1009071)

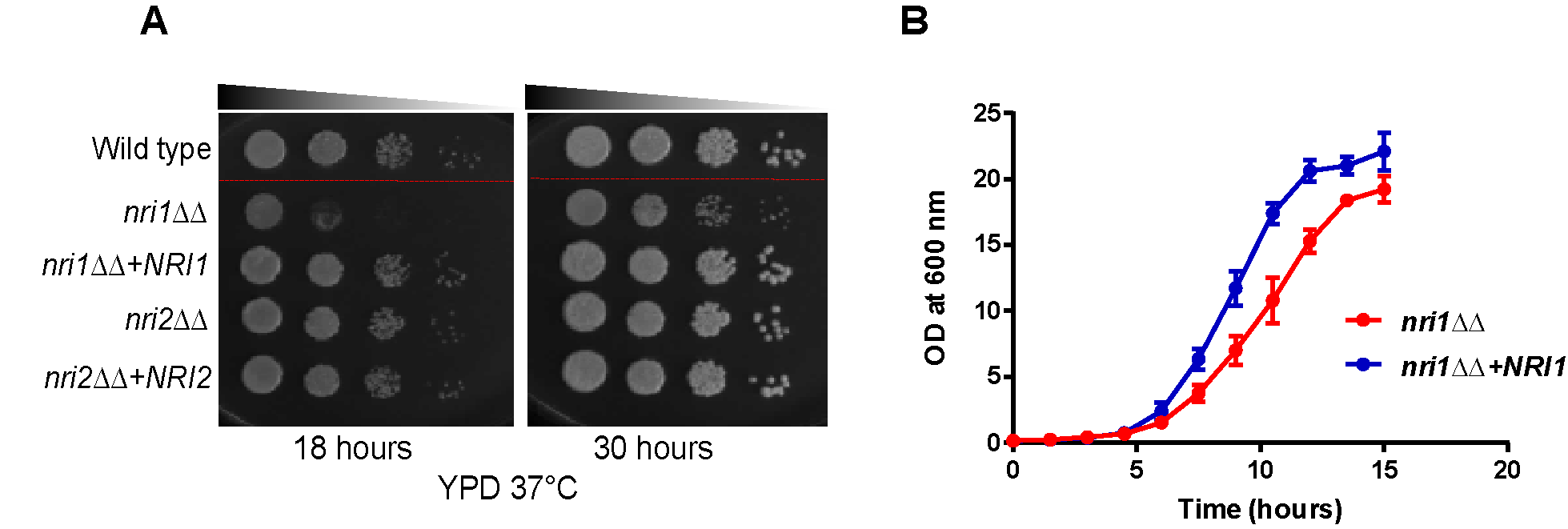

Supplement: S1 Fig — A. Growth of the indicated strains on YPD plates, photographed after 18 and 30 hours of incubation at 37°C. (Red dotted line indicates cropping within the same image). B. Growth curve of the indicated strains in YPD at 30°C. Absorbance at 600 nm was recorded every 90 minutes. Mean values with standard deviation from three independent experiments were plotted. Raw data underlying graph given in S7 Table. (TIF) [file pgen.1009071.s001.tif]

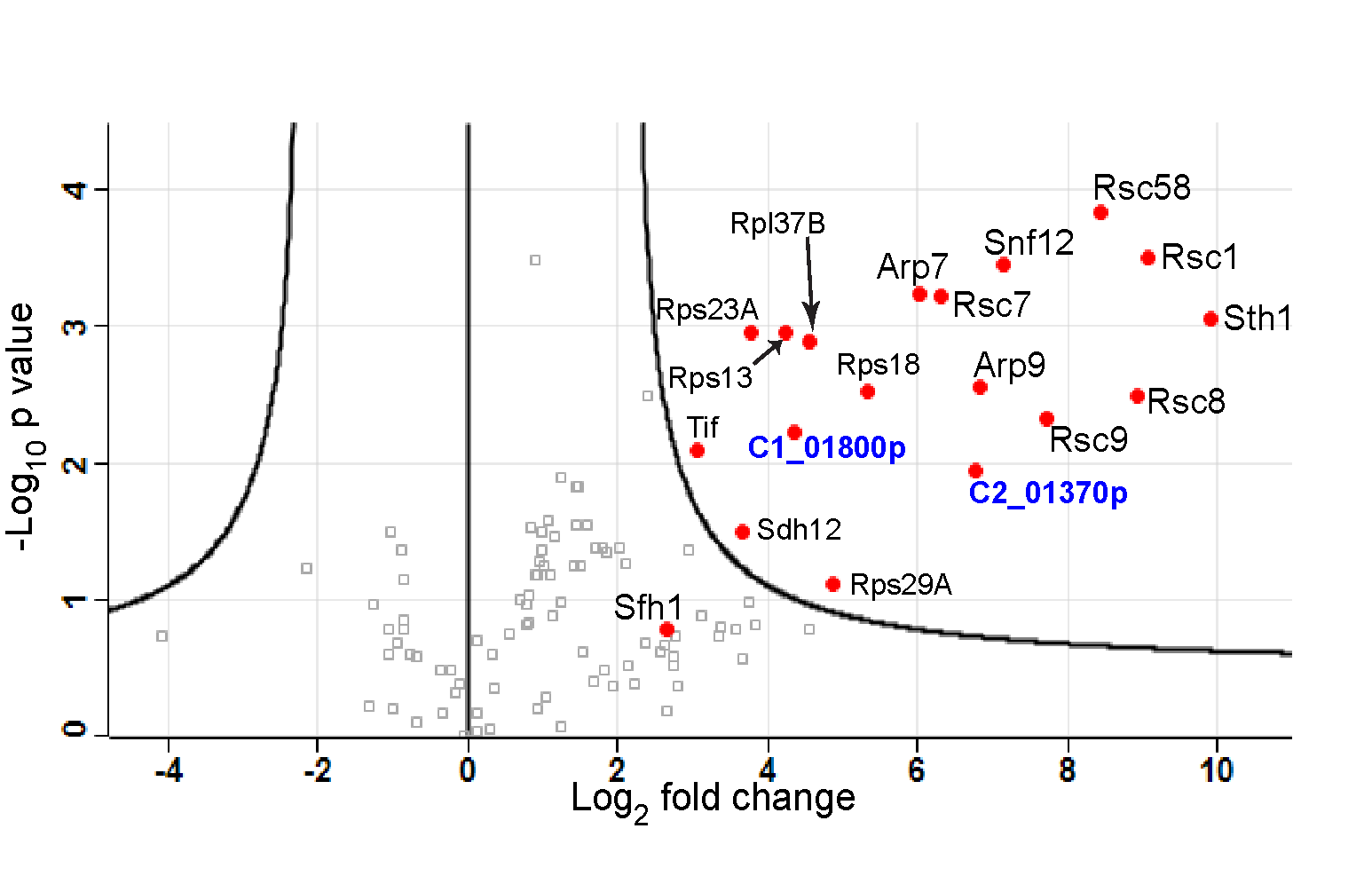

Supplement: S2 Fig — Volcano plot representation of significantly enriched proteins that copurified with Sth1-Myc in rsc4ΔΔ (SGC2011) compared to a no-tag control (SGC2006). Significant interactors identified are shown on the right side of the parabola as red dots, except for one potential interactor with low confidence score (Sfh1), highlighted on the left side. The grey boxes represent the potential background interactors. The two novel interactors are labelled in blue. The x-axis denotes the enrichment of the interaction in Sth1-Myc in rsc4ΔΔ samples with respect to no-tag control, represented as a natural logarithm of normalized intensity values. The negative logarithm to the base 10 of the p-value, based on Student’s t-test, is given on the y-axis. (TIF) [file pgen.1009071.s002.tif]

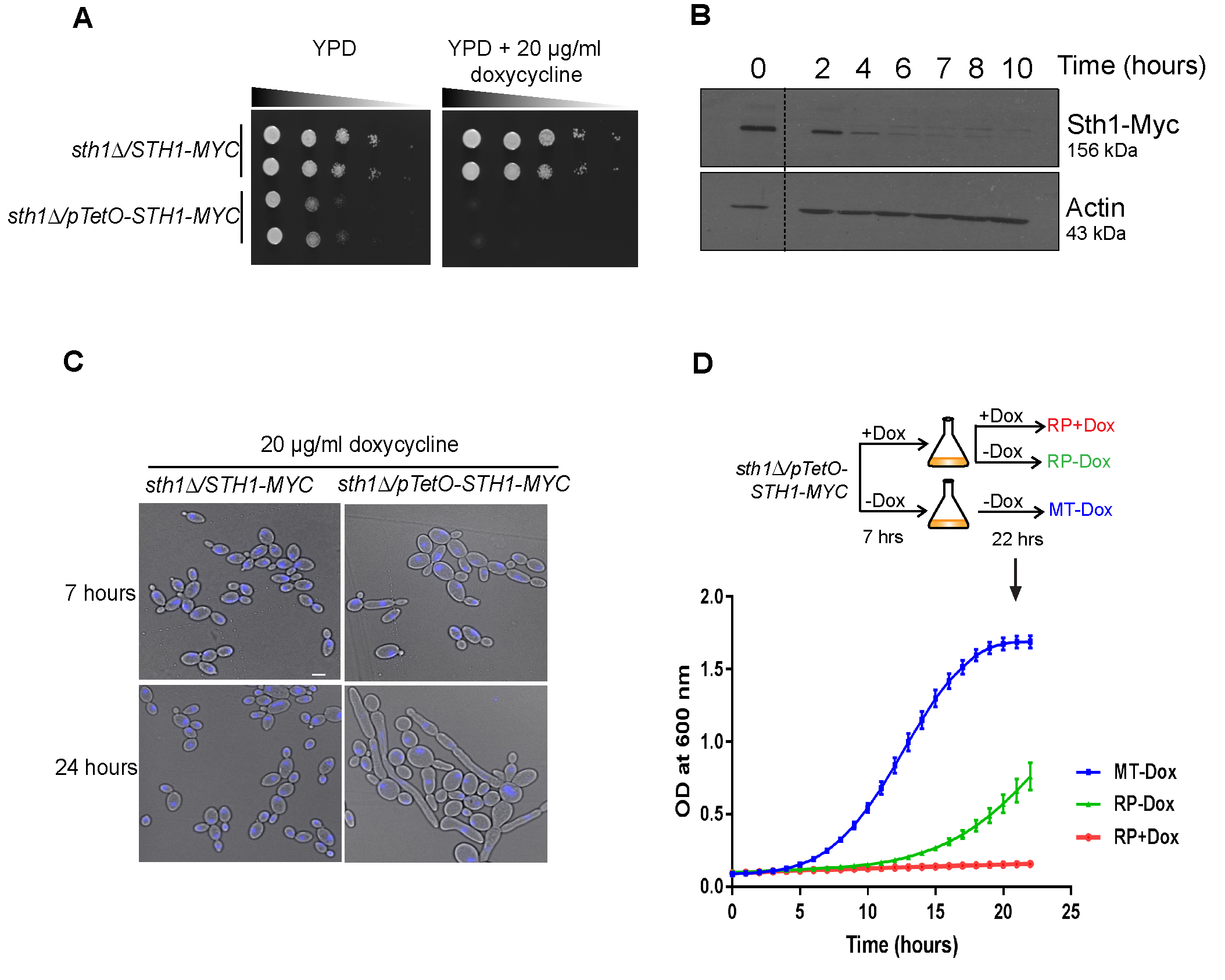

Supplement: S3 Fig — A. Two independent clones of hemizygous and doxycycline-repressible conditional sth1 mutants (sth1Δ/STH1-MYC:SGC2008; sth1Δ/pTetO-STH1-MYC:SGC2001) were spotted on YPD or YPD containing 20 μg/ml doxycycline (dox) plates. After 30 hours of growth at 30°C, the plates were photographed. B. Conditional sth1 mutant (sth1Δ/pTetO-STH1-MYC) cells were treated with 20 μg/ml dox for the indicated time period and Sth1-Myc levels were monitored by immunoblotting using anti-myc antibody. Actin was used as the loading control and detected using anti-actin monoclonal antibody. C. Cellular morphology of hemizygous and conditional sth1 mutants treated with 20 μg/ml dox for 7 and 24 hours respectively (bar-5 μm). D. Schematic showing the strategy to assess the viability of conditional sth1 mutant treated with dox for 7 hours. sth1Δ/pTetO-STH1-MYC cells were initially cultured in the presence or absence of dox for 7 hours. Dox-treated cells were then split and re-passaged in media containing dox (RP+Dox) or lacking dox (RP-Dox). The untreated cells were re-passaged only in dox-lacking media (MT-Dox). The growth profiles of the cells in these cultures monitored in 96-well plates for 22 hours at 30°C with shaking at 200 rpm, are given below the schematic. Mean values with standard deviation are shown from 3 experiments. Raw data underlying graph given in S7 Table. (TIF) [file pgen.1009071.s003.tif]

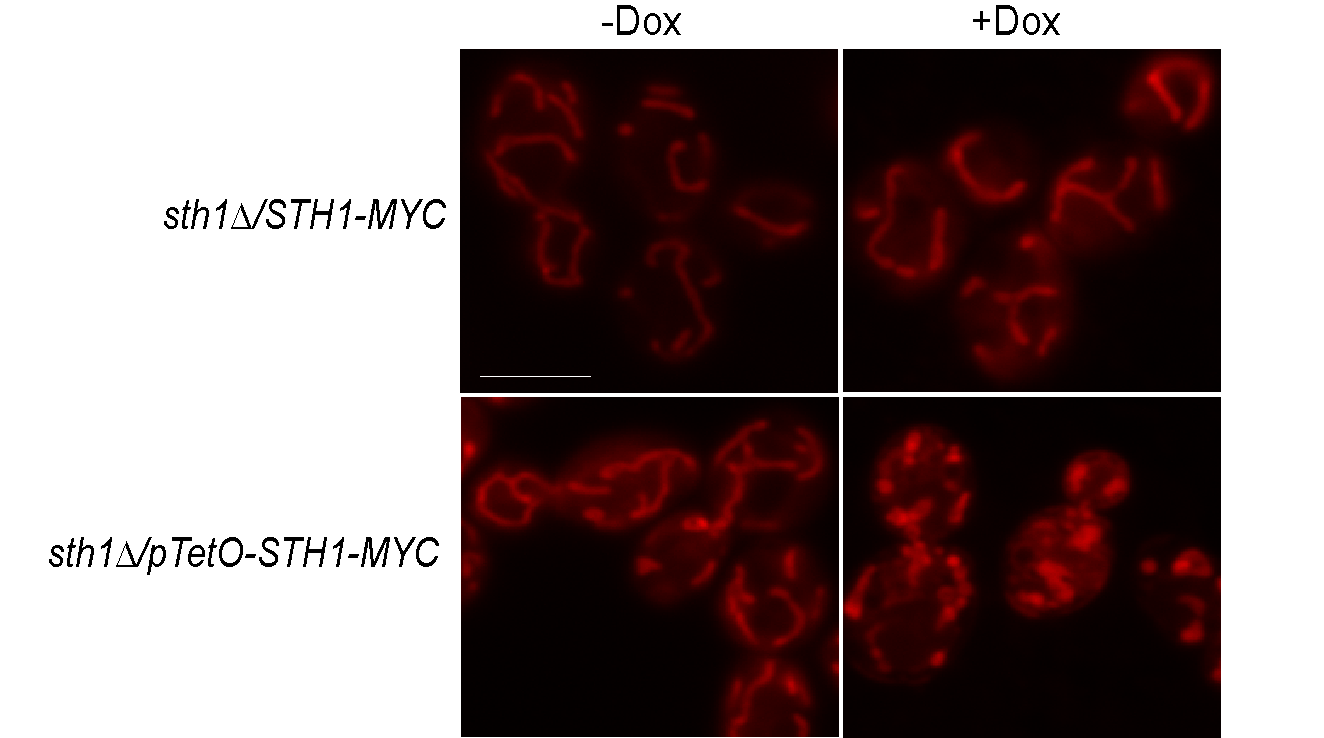

Supplement: S4 Fig — Mitochondrial morphology assessed by MitoTracker staining of the indicated cells (sth1Δ/STH1-MYC:SGC2008; sth1Δ/pTetO-STH1-MYC:SGC2001) cultured with or without dox for 7 hours in YPD at 30°C with shaking at 200 rpm (bar—5 μm). (TIF) [file pgen.1009071.s004.tif]

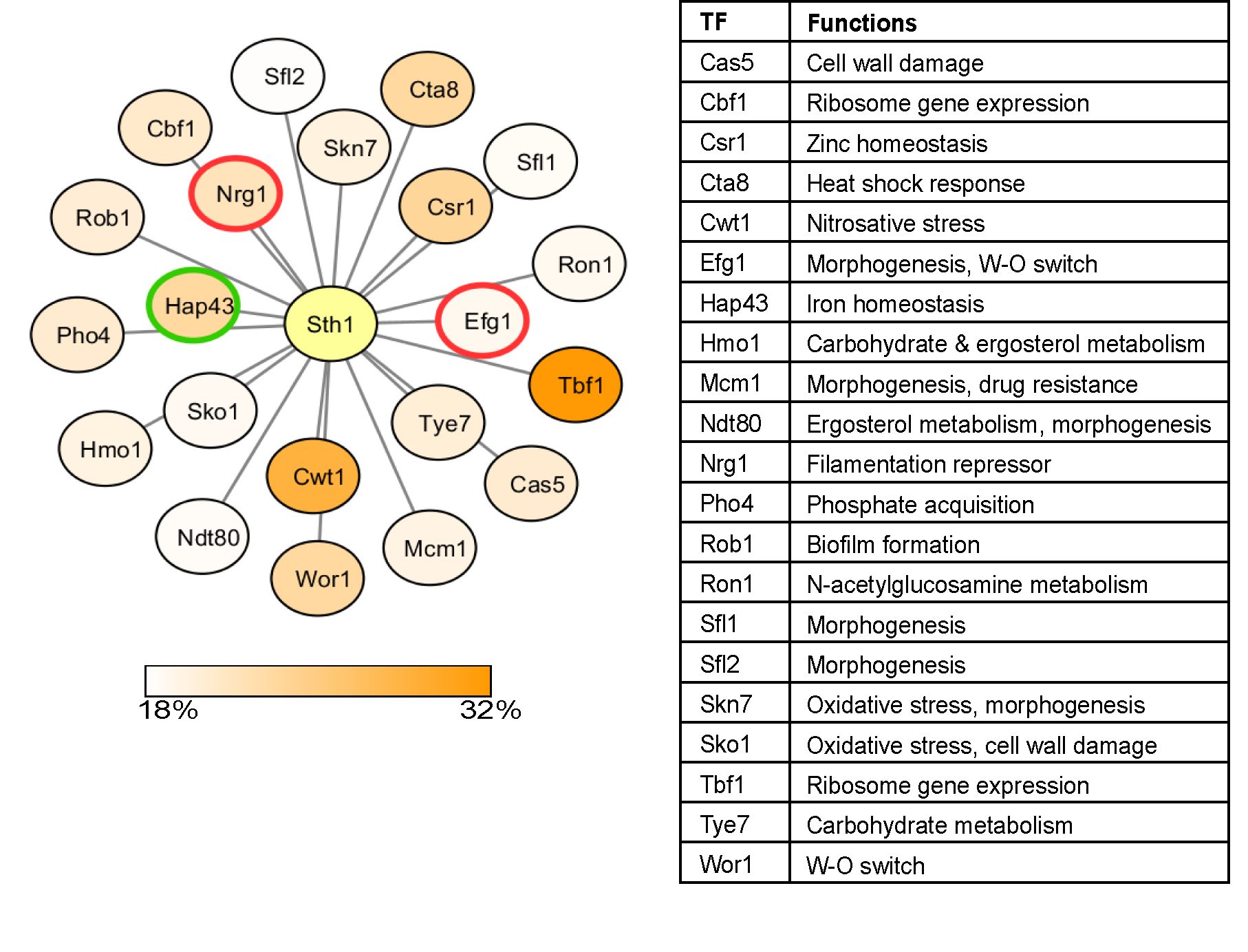

Supplement: S5 Fig — The transcription factors (TFs) associated with sth1 transcriptome profile identified from PathoYeastract database (analysis performed on 8 June 2019). Differentially expressed genes filtered by 1.5-fold change were analyzed and represented as an interaction network using Cytoscape. The circles other than Sth1, are shaded based on the percent of TF targets enriched in the dataset, as indicated by a color scale at the bottom. In addition, TFs mis-regulated in the sth1 RNA-Seq dataset (1.5-fold change cutoff) are highlighted with a red border (if up-regulated) or green border (if down-regulated). A table mentioning the functions of the enriched TFs based on published literature is given on the right. Raw data underlying graph given in S7 Table. (TIF) [file pgen.1009071.s005.tif]

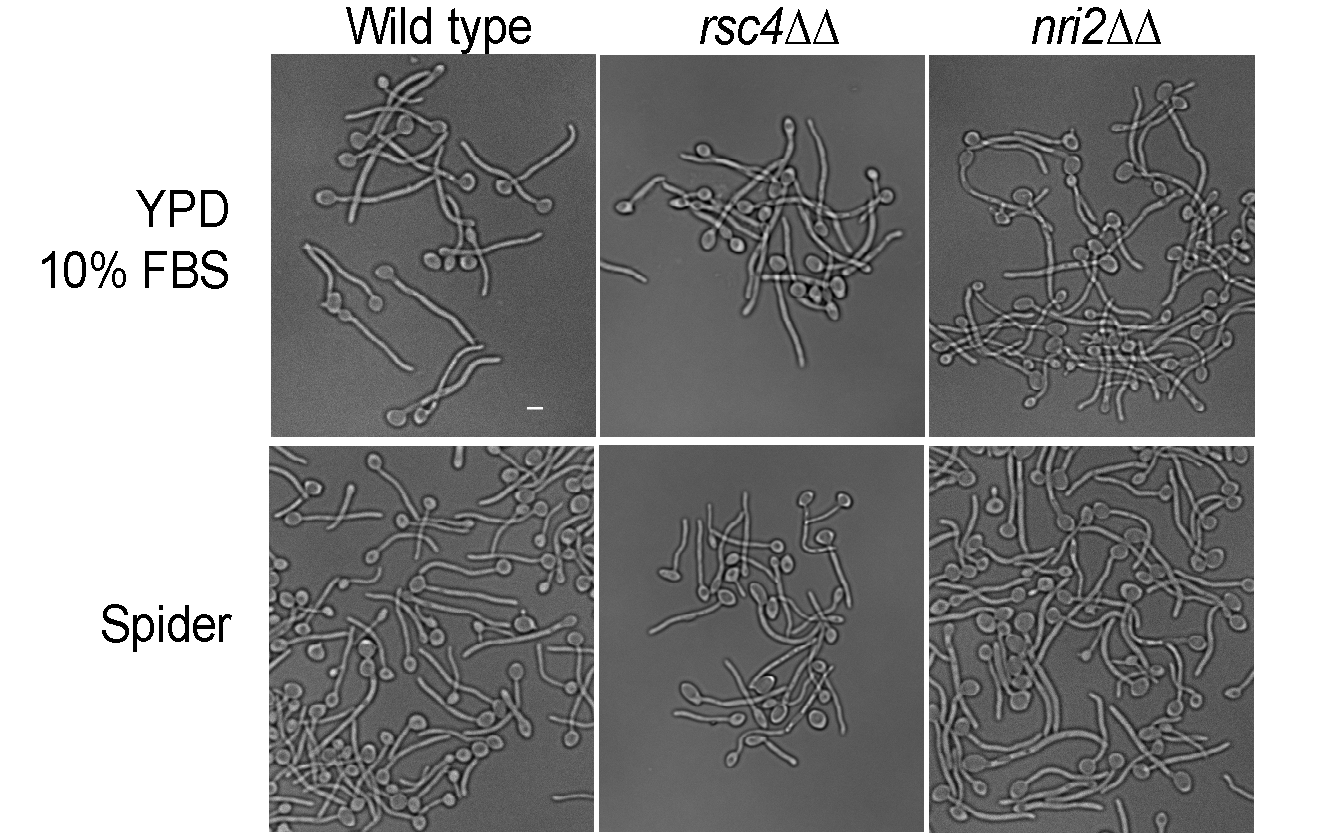

Supplement: S6 Fig — Cellular morphology of wild type (SGC2024), rsc4ΔΔ (SGC2022) and nri2ΔΔ (SGC2032) induced to form hyphae in the indicated liquid media. Images were acquired after 90 minutes of induction at 37°C (bar-5 μm). (TIF) [file pgen.1009071.s006.tif]

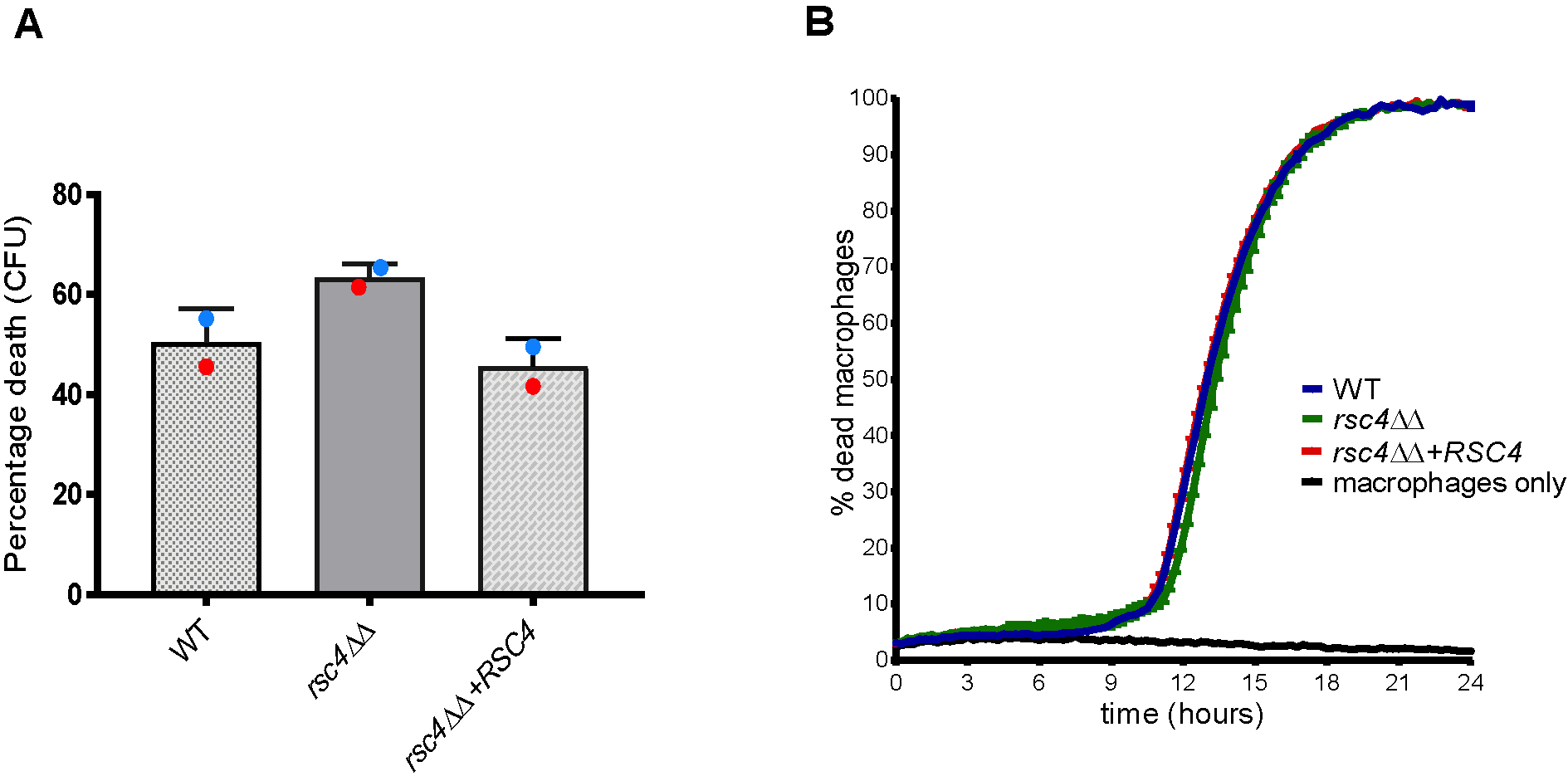

Supplement: S7 Fig — A. Intracellular killing of wild type (SGC2024), rsc4ΔΔ (SGC2022) and re-integrant (SGC2023) by human neutrophils (MOI 1) post 1 hour incubation, assessed by CFU counting. Percent death relative to no neutrophil control was plotted for each strain. Red and blue dots indicate percent values from two independent experiments and error bars denote standard deviation. Raw data underlying graph given in S7 Table. B. Kinetics of macrophage death in the presence of wild type (SGC2024), rsc4ΔΔ (SGC2022) and re-integrant (SGC2023) cells (MOI 1) assessed by live cell imaging for 24 hours. Mean values with SEM from two biological replicates were plotted, with at least 5000 macrophages surveyed for each strain. Raw data underlying graph given in S7 Table. (TIF) [file pgen.1009071.s007.tif]

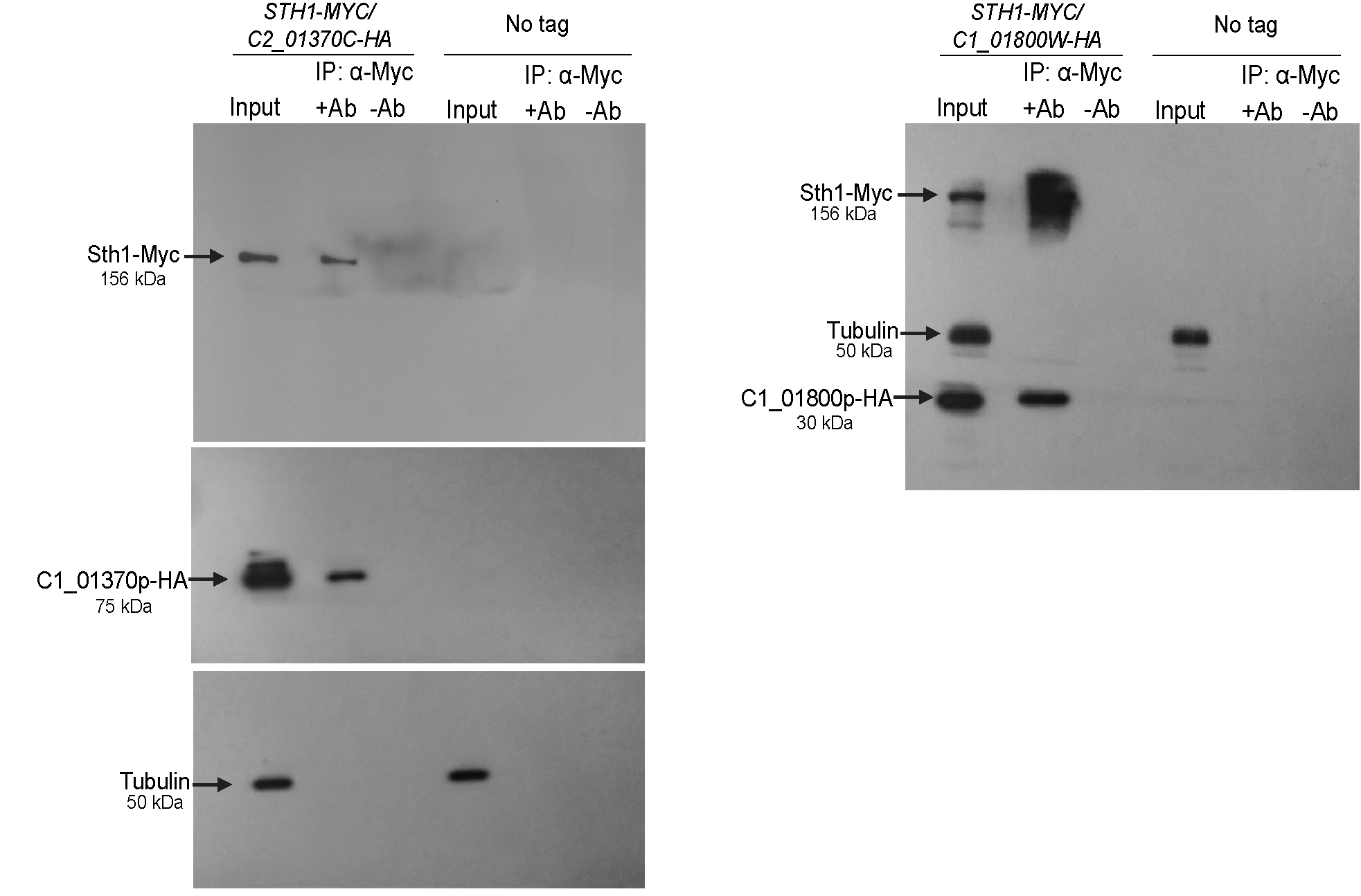

Supplement: S8 Fig — Cell lysates from tagged and no tag control strains were immunoprecipitated with polyclonal anti-myc (ab9106) antibody. Sth1-Myc and Nri1-HA (C2_01370p-HA) or Nri2-HA (C1_01800p-HA) were detected by immunoblotting using monoclonal anti-Myc (9E10) and anti-HA (3F10) antibodies, respectively. Tubulin was used as the loading control. Two percent of input and 50% of eluate were loaded. (TIF) [file pgen.1009071.s008.tif]

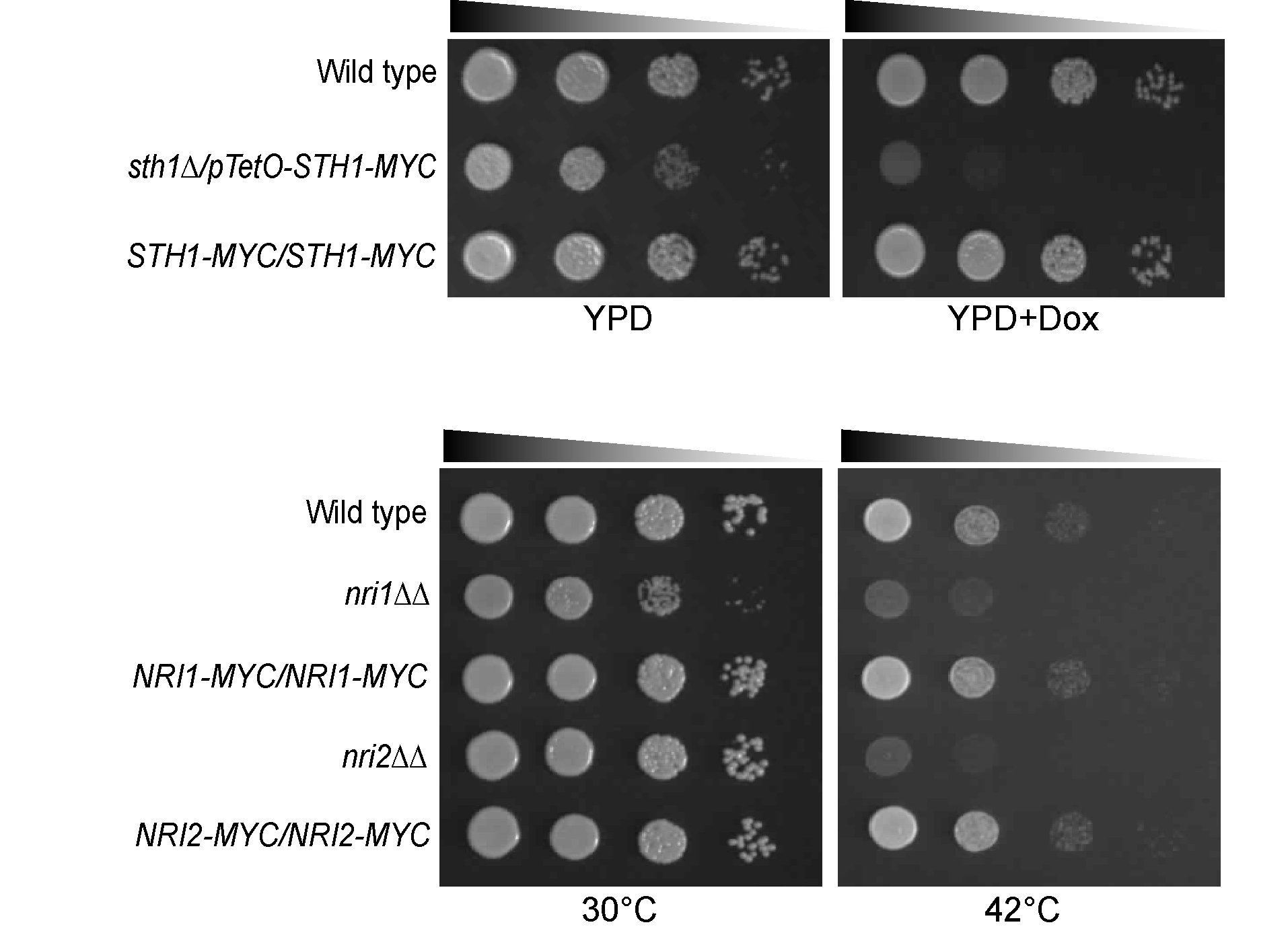

Supplement: S9 Fig — Top panel shows the growth of wild type (SGC2006), sth1Δ/pTetO-STH1-MYC (SGC2001) and STH1-MYC/STH1-MYC (SGC2003) cells spotted on YPD or YPD containing 20 μg/ml dox plates. Bottom panel shows the growth of wild type (SGC2006), MYC-tagged NRI1 (SGC2017) and NRI2 (SGC2019), and their respective null mutants (nri1ΔΔ:SGC2047; nri2ΔΔ:SGC2032) at 30°C and 42°C. Images were taken after 2 days of incubation. (TIF) [file pgen.1009071.s009.tif]
